# Single‐time‐point renal dosimetry for [^177^Lu]Lu‐PSMA‐617: Incorporating multi‐cycle population data using nonlinear mixed‐effects models

**DOI:** 10.1002/mp.70736

**Published:** 2026-10-22

**Authors:** Indra Budiansah, Steffie M. B. Peters, Bastiaan Privé, James Nagarajah, Mark W. Konijnenberg, Gerhard Glatting, Deni Hardiansyah

**Affiliations:** ^1^ Medical Physics and Biophysics Division Physics Department Faculty of Mathematics and Natural Sciences Universitas Indonesia Depok Indonesia; ^2^ Medical Radiation Physics Department of Nuclear Medicine Ulm University Ulm Germany; ^3^ Department of Medical Imaging Radboud University Medical Center Nijmegen The Netherlands; ^4^ Department of Radiology and Nuclear Medicine Erasmus Medical Center Rotterdam The Netherlands; ^5^ Röntgeninstitut Düsseldorf Düsseldorf Germany; ^6^ Department of Radiation Oncology Erasmus Medical Center Rotterdam The Netherlands

**Keywords:** [^177^Lu]Lu‐PSMA‐617, multi‐cycle therapy, NLME modeling, NONMEM, STP dosimetry, TIACs

## Abstract

**Background:**

Single‐time‐point (STP) dosimetry using nonlinear mixed‐effects (NLME) modeling has emerged as a promising approach, with recent studies demonstrating high accuracy. This framework enables estimation of the time‐integrated activity coefficient (TIAC) from a single imaging time point and, consequently, prediction of absorbed dose for subsequent therapy cycles. However, the use of multi‐cycle population data to support NLME‐based STP dosimetry has not yet been systematically evaluated.

**Purpose:**

This study aimed to evaluate whether incorporating multi‐cycle population data into an NLME framework improves agreement between STP‐derived TIACs and all‐time‐point (ATP)‐derived model‐based reference TIACs in renal dosimetry.

**Methods:**

Renal biokinetic data [^177^Lu]Lu‐PSMA‐617 were collected from 10 patients over two therapy cycles, with both kidneys analyzed, yielding 40 renal datasets. In both cycles, SPECT/CT imaging was performed at five time points. A literature‐based structural function was used in the NLME modeling to incorporate inter‐individual variability, inter‐cycle variability, and variability between left and right kidney positions. Goodness‐of‐fit was evaluated based on visual inspection, relative standard errors (RSEs) < 50%, and the absolute value of the off‐diagonal elements in the correlation matrix < 0.8. TIACs derived from ATP fitting were used as the reference (rTIACs). STP fitting was performed using the leave‐one‐out method across defined scenarios. The TIACs derived from STP fitting were designated as STP TIACs (sTIACs). The accuracy of sTIACs in predicting rTIACs was assessed using relative deviations (RDs) and mean absolute percentage error (MAPE).

**Results:**

The model fits satisfied all predefined goodness‐of‐fit criteria. STP dosimetry using the multi‐cycle dataset offered greater flexibility in time‐point combinations, leading to MAPEs < 10%. Additionally, an improvement in early‐time‐point STP dosimetry was observed when using multi‐cycle data.

**Conclusions:**

Incorporating multi‐cycle data into NLME‐based STP dosimetry reduced extreme RDs, increased flexibility in feasible time‐point combinations, and improved early‐time‐point STP performance in this small two‐cycle cohort.

## 1 INTRODUCTION

Accurate and precise estimation of time‐integrated activity coefficients (TIACs) is essential in radiopharmaceutical therapy (RPT). These coefficients are important for estimating absorbed doses, which in turn are critical for ensuring both treatment safety and therapeutic efficacy.^1^, ^2^ The conventional approach to TIAC estimation relies on serial imaging, requiring the acquisition of patient‐specific biokinetic data at multiple time points following radiopharmaceutical administration.^3^ The importance of sampling‐time optimization has also been demonstrated in [^177^Lu]Lu‐PSMA dosimetry simulations, where the number and timing of measurements affected the accuracy and precision of renal and tumor TIAC estimates.^4^ However, these serial imaging protocols require significant clinical resources, contributing to patient burden (e.g., prolonged immobilization, repeated biokinetic measurements) and to the clinical workload (e.g., imaging, data analysis).^5^, ^6^, ^7^ These challenges have driven interest in single‐time‐point (STP) dosimetry approaches.^7^, ^8^, ^9^, ^10^, ^11^, ^12^, ^13^, ^14^, ^15^, ^16^

STP dosimetry has been increasingly investigated across various radiopharmaceuticals, with numerous studies proposing different methods to improve its accuracy.^8^, ^9^, ^11^, ^13^, ^15^ Early investigations, such as those by Hänscheid et al.^8^ and Madsen et al.,^9^ which assumed monoexponential biokinetics, demonstrated that STP dosimetry within a specific time range could yield acceptable TIAC estimates as evidenced in [^177^Lu]Lu‐DOTA‐TATE/TOC and [^90^Y]Y‐DOTA‐TOC therapies. Recently, we proposed the use of population‐based model selection with nonlinear mixed‐effects (PBMS NLME) modeling in [^90^Y]Y‐DOTA‐TATE and [^177^Lu]Lu‐PSMA‐617 therapies^11^, ^13^, ^16^ for STP dosimetry, with the structural fit function selected based on the Akaike weight.^17^, ^18^ Unlike conventional methods that presuppose monoexponential biokinetics,^7^, ^8^, ^9^, ^15^ PBMS NLME modeling selects the optimal fit function based on goodness‐of‐fit test criteria^19^, ^20^, ^21^ and Akaike weights,^17^, ^18^, ^19^, ^20^, ^21^ thereby ensuring that the selected function is most supported by the dataset. This data‐driven selection is particularly crucial given that arbitrary variations in time‐activity curve fitting methods have been identified as a major source of dosimetric variability in RPT.^22^ A recent study extended this PBMS NLME concept to few‐time‐point renal dosimetry for [^177^Lu]Lu‐PSMA‐617 and showed that NLME‐based modeling analysis can improve both accuracy and precision as additional time points are incorporated.^23^ This supports the broader premise that sparse‐data dosimetry should be evaluated not only for bias but also for precision and robustness.

Previous studies^13^, ^16^ have demonstrated that STP dosimetry using PBMS NLME modeling outperforms the commonly used monoexponential fit function.^8^, ^9^ These findings suggest that NLME‐based STP methods can provide more accurate estimates of TIACs than the frequently used monoexponential biokinetic assumption, reducing the root‐mean‐square error (RMSE) at the optimal time points from 34% to 10%.^13^ Nevertheless, despite their advantages, NLME‐based STP dosimetry using early time points can still yield substantial errors. For example, in renal STP dosimetry for [^1^ ^7^ ^7^Lu]Lu‐PSMA‐617, deviations ranging from −55% to 59% have been reported when early post‐injection time points are used.^13^ These observations underscore the need for strategies that mitigate large estimation errors and reduce the occurrence of extreme values in early‐time‐point STP dosimetry.

RPT is commonly performed over multiple therapy cycles.^14^, ^15^, ^24^ However, implementations of STP dosimetry using PBMS NLME modeling have been applied exclusively to single‐cycle therapies.^11^, ^12^, ^13^, ^16^ Although using a different method, Willowson et al.^15^ have demonstrated that STP dosimetry using individual fitting incorporating data from multi‐cycle therapy may enhance the accuracy of STP dosimetry results. Integrating biokinetic data from multi‐cycle therapies into NLME modeling may improve model robustness by capturing more variation in inter‐individual, inter‐cycle, and residual variability in multiple therapy cycles.^25^, ^26^ This raises two research questions: (a) Does using multi‐cycle population data within the NLME modeling improve the accuracy of TIAC estimation in STP dosimetry? and (b) How does STP accuracy vary across time‐point combinations in multi‐cycle therapy? To date, no study has systematically addressed these gaps. Therefore, this study aims to evaluate the performance of STP dosimetry when multi‐cycle datasets are incorporated and investigate the cycle‐specific accuracy of STP TIAC estimates.

## 2 MATERIALS AND METHODS

### 2.1 Biokinetic data

Biokinetic data of [^177^Lu]Lu‐PSMA‐617 in the left and right kidneys were obtained from 10 patients diagnosed with low‐volume metastatic hormone‐sensitive prostate cancer (mHSPC) enrolled in a prospective clinical study (NCT03828838).^27^ Each patient had left‐ and right‐kidney biokinetic measurements in each cycle, resulting in 40 renal datasets. Left and right kidneys were analyzed separately because absorbed‐dose assessment in renal dosimetry is organ‐specific and because the analysis should not assume complete left‐right symmetry a priori. However, both kidneys originate from the same patient and are therefore not statistically independent; this dependence should be considered when interpreting performance metrics and variability estimates.

All patients received two cycles of [^177^Lu]Lu‐PSMA‐617 therapy with an 8‐week interval between cycles. The injected activity was (3.1 ± 0.1) GBq in the first cycle (C1) and (5.9 ± 0.4) GBq in the second cycle (C2). Three‐bed‐position SPECT/CT imaging was performed over the pelvis, abdomen, and head–neck regions. Five SPECT/CT acquisitions were performed in both cycles for each patient at the following time points after injection (mean ± SD): TP1 (2.5 ± 0.9) h, TP2 (23 ± 2) h, TP3 (47 ± 2) h, TP4 (71 ± 2) h, and TP5 (167 ± 2) h. The acquisition and reconstruction protocol adhered to the approach reported by Peters et al.^28^ and aligned with the recommendations of MIRD Pamphlet No. 26.^29^ These protocols include scatter, attenuation, and dead‐time corrections. A comprehensive description of image acquisition and quantification methods has been provided in previously published studies.^28^, ^30^

### 2.2 Reference model

The structural fit function for the reference in this study is a sum‐of‐exponentials function (SOEF) adapted from a previous study.^13^ That study obtained the function based on modeling renal biokinetic data from 63 patients with metastatic castration‐resistant prostate cancer (mCRPC) in the first cycle of [^177^Lu]Lu‐PSMA‐617 therapy with five‐time‐point SPECT/CT imaging ranging from 1 h up to 1 week after injection. The SOEF is defined as in Equation (1):

(1) $$\begin{array}{cc} f\left(t\right) & =A_{1}\;e^{-\left(\lambda_{1}+\lambda_{\mathrm{phys}}\right)t}+A_{2}e^{-\left(\lambda_{2}+\lambda_{\mathrm{phys}}\right)t}\\ & \;-A_{3}e^{-\left(\lambda_{3}+\lambda_{\mathrm{phys}}\right)t}-\left(A_{1}+A_{2}-A_{3}\right)e^{-\left(\lambda_{\mathrm{bc}}+\lambda_{\mathrm{phys}}\right)t} \end{array}$$

where $A_{j}\left(j=1,2,3\right)$ are the coefficients of the respective exponential term; and $\lambda_{j}\left(j=1,2,3\right)$ are the biological uptake or clearance rates of the [^177^Lu]Lu‐PSMA‐617 radiopharmaceutical in the kidneys. In addition, the rapid uptake of the radiopharmaceutical due to blood circulation was added and assumed to possess a half‐life of one minute $\left(\;\lambda_{\mathrm{bc}}=\frac{\ln2}{1\;\min}\;\right)$.^13^, ^16^, ^31^ The model was fitted simultaneously to all biokinetic data using NLME modeling, implemented in NONMEM (version 7.6.0, ICON Development Solutions, Ellicott City, MD).^32^

In NLME modeling, individual parameters are described as a combination of fixed and random effects. The fixed effects represent the typical population parameter values, whereas the random effects in this study describe inter‐individual variability (IIV), left‐right kidney position variability, inter‐cycle variability, and residual variability. Both fixed and random effects were estimated from the dataset. This study employs the exponential variability model to describe the individual parameters, as it ensures positive parameter values and is commonly used to describe pharmacokinetic parameters.^25^, ^26^, ^33^ The individual parameters in this study can be expressed as:

(2) $$\theta_{kipq}=\theta_{k}e^{\eta_{ki}+\varphi_{kip}+\kappa_{kipq}}$$

(3) $$\eta_{ki}∼N\left(0,\omega^{2}\right);\varphi_{kip}∼N\left(0,\Gamma^{2}\right);\kappa_{kipq}∼N\left(0,\Pi^{2}\right)$$

where $\theta_{kipq}$ is the parameter k of individual i for kidney p (left/right) in cycle q; $\theta_{k}$ denotes the typical value of parameter k, estimated for the population; $\eta_{ki}$ represents individual‐specific deviation from $\theta_{k}$ (in log‐domain); $\varphi_{kip}$ represents the variability of the parameter due to differences between the left and right kidney positions and $\kappa_{kipq}$ describes the variability of parameter k in cycle q. The random effects $\eta_{ki}$, $\varphi_{kip}$, and $\kappa_{kipq}$ are normally distributed with a mean of zero and variance of $\omega^{2}$, $\Gamma^{2}$, and $\Pi^{2}$, respectively. During the model development, all parameters were assumed to have random effects. After the first minimization, the parameters exhibiting negligible variability (i.e., variance < 10^−6^) were assigned a variance of 0.^34^ A combined inter‐individual and inter‐cycle random‐effect structure was also investigated because the predefined variability components could not be estimated independently due to the small cohort size.

Residual variability, referring to differences between model predictions and measured biokinetic data, was described using the proportional error model^25^, ^26^ as

(4) $$Y=Y_{\mathrm{pred}}\;\left(1+\varepsilon \right)$$

(5) $$\varepsilon ∼N\left(0,\sigma^{2}\right)$$

where Y is the measured biokinetic data; $Y_{\mathrm{pred}}$ describes the predicted biokinetic data from the fit function and ε is the residual error, which is assumed to be normally distributed with a mean of zero and variance of $\sigma^{2}$. The goodness‐of‐fit criteria applied in ATP fitting were visual inspection of the biokinetic curves, relative standard errors (RSEs) of the fitted parameters < 50%, and absolute values of the off‐diagonal elements of the correlation matrix < 0.8.^19^, ^20^, ^21^ Additionally, the NLME modeling goodness‐of‐fit assessment, including the conditional weighted residual (CWRES)^35^ and the visual predictive check (VPC), was added to provide the model assessment within the NLME modeling framework.^36^ The reference TIACs (rTIACs) were calculated analytically by integrating the SOEF (Equation 1) from time zero to infinity with the estimated parameters derived from all‐time‐point (ATP) biokinetic data. The rTIACs were used as model‐based reference estimates for method comparison. They should not be interpreted as true TIACs, but as the best available reference estimates derived from all available time points using the predefined structural modeling framework. This study defined four modeling scenarios to evaluate the STP performance in predicting rTIACs using STP biokinetic data. The summary of the four defined scenarios is presented in Figure 1.

**FIGURE 1:**
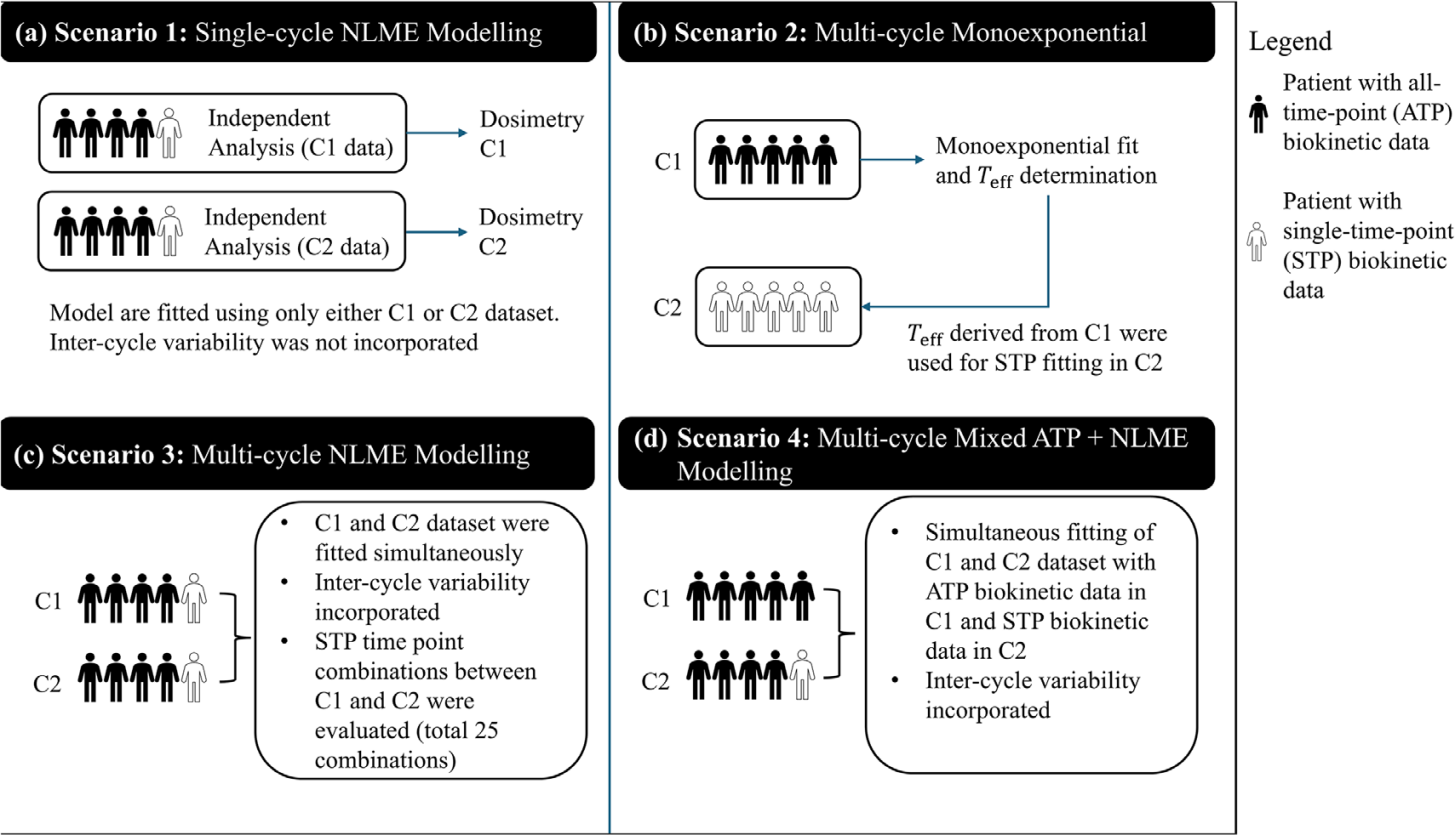
Defined scenarios of single‐time‐point (STP) dosimetry evaluation. This figure illustrates the four modeling scenarios investigated to evaluate the performance of single‐time‐point (STP) dosimetry in predicting time‐integrated activity coefficients (TIACs) derived from all‐time‐point (ATP) biokinetic data. Across all NLME modeling scenarios, STP dosimetry was performed using a leave‐one‐out approach: each patient was iteratively fitted using only their STP biokinetic data, while the remaining patients in the cohort were fitted using their ATP data. **(a)** Scenario 1: single‐cycle nonlinear mixed‐effects (NLME) modeling. STP dosimetry is performed independently within a single cycle, either the first cycle (C1) or the second cycle (C2), employing the biokinetic data from the corresponding cycle within the NLME modeling framework. **(b)** Scenario 2: multi‐cycle monoexponential fitting. The effective half‐life is initially derived by fitting a monoexponential function to the ATP biokinetic data of C1 using individual‐based fitting. This C1‐derived effective half‐life is subsequently employed in C2 to fit the STP biokinetic data in the subsequent cycle.^15^ **(c)** Scenario 3: multi‐cycle NLME modeling. STP fitting was performed simultaneously across both C1 and C2 using the full dataset within the NLME framework, except that the investigated patient was fitted using STP biokinetic data. Additionally, combinations of STP time points between C1 and C2 were explored. **(d)** Scenario 4: ATP data in C1 and STP data in C2 within the NLME modeling. This scenario uses a combined dataset where C1 includes ATP data and C2 employs STP data. The NLME model is fitted using biokinetic data from both cycles, with STP fitting exclusively performed in C2.

### 2.3 Scenario 1: single‐cycle dataset for single‐time‐point dosimetry with nonlinear mixed‐effects modeling

In this scenario, STP dosimetry was performed using only a single‐cycle dataset (either C1 or C2 dataset).^13^ Therefore, the inter‐cycle variability (represented by $\kappa_{kipq}$ in Equation 2) was not considered. The structural fit function is Equation (1). The patient‐based leave‐one‐out method was employed for this STP dosimetry approach, i.e., the SOEF was fitted simultaneously across all patients using NLME modeling, and this fitting used ATP biokinetic data from all patients except the one under investigation, for whom only an STP biokinetic datum was used. This patient‐based leave‐one‐out method was then replicated for every patient and every time point. In each leave‐one‐out iteration, the left‐out unit was the patient. Thus, all kidney and cycle measurements from the investigated patient were withheld from the population‐model component, except for the STP or ATP measurements explicitly allowed for that scenario. This ensured that ATP information from the same patient was not inadvertently used to support the STP estimate unless it was part of the predefined scenario design. The resulting TIACs derived from STP fitting were denoted as STP TIACs (sTIACs).

### 2.4 Scenario 2: Monoexponential individual‐based fitting

Willowson et al.^15^ demonstrated that in [^177^Lu]Lu‐DOTA‐TATE therapy, STP dosimetry for the second and subsequent cycles, performed assuming a monoexponential fit function with individual‐based fitting, yields acceptable TIAC estimates. In this method, the ATP biokinetic data in C1 were individually fitted using a monoexponential function. The effective half‐life derived from C1 was then used in the following cycle, allowing the prefactor of the monoexponential function to be fitted using an STP biokinetic measurement.

### 2.5 Scenario 3: Multi‐cycle dataset within nonlinear mixed‐effects modeling

STP dosimetry was performed employing a multi‐cycle dataset and the patient‐based leave‐one‐out method (as performed in Scenario 1). In this scenario, the inter‐cycle variability (represented by $\kappa_{kipq}$ in Equation 2) was incorporated to account for the variability between therapy cycles, and a combined IIV and inter‐cycle variability was also investigated. Additionally, STP dosimetry with time‐point combinations across two cycles was investigated. For instance, STP dosimetry in C1 was performed at TP1 (2.5 ± 0.9) h, while C2 was assessed from TP1 (2.5 ± 0.9) h to TP5 (167 ± 2) h, yielding a total of 25 time‐point combinations between the two cycles for each patient.

### 2.6 Scenario 4: All‐time‐point dosimetry in the first cycle and single‐time‐point dosimetry for the following cycle within nonlinear mixed‐effects modeling

In this scenario, multi‐cycle data were used, and inter‐cycle variability and a combined IIV and inter‐cycle variability were incorporated as in Scenario 3. The patient‐based leave‐one‐out method was employed: all patients were fitted using ATP biokinetic data from both C1 and C2, except for the patient under investigation. For the investigated patient in Scenario 4, the ATP data from C1 were intentionally retained as prior‐cycle patient‐specific information, whereas C2 was represented only by the selected STP measurement. No additional C2 time points from that patient were used for estimating the C2 sTIAC.

### 2.7 Performance metrics and scenario evaluations of single‐time‐point dosimetry

The accuracy of sTIACs for predicting rTIACs in each scenario was evaluated using relative deviations (RDs), mean absolute percentage error (MAPE), and RMSE. The RD was calculated as follows:

(6) $${\mathrm{RD}}_{i}=\frac{\mathrm{sTIA}C_{i}-\mathrm{rTIA}C_{i}}{\mathrm{rTIA}C_{i}}\;$$

with i being the index of the i‐th kidney dataset. To visualize the distribution and variance of the predictive errors across different scenarios, the RDs were summarized using boxplots. In these visualizations, the central horizontal line denotes the median RD, while the lower and upper hinges of the box represent the first (25th percentile) and third (75th percentile) quartiles, respectively. The whiskers extend from the hinges to the most extreme data points that fall within 1.5 × interquartile range (IQR). Individual points plotted beyond the whiskers represent outlier values.

The MAPE was calculated according to Equation (7):

(7) $$\mathrm{MAP}E_{j}=\frac{1}{n}\;\sum_{i=1}^{n}\left|RD_{ij}\right|$$

where $\mathrm{MAP}E_{j}$ is the MAPE at time point j, n is the total number of investigated kidneys, and $RD_{ij}$ denotes the relative deviation for patient/kidney dataset i at time point j. The RMSE was calculated according to Equation (8):

(8) $$\mathrm{RMS}E_{j}=\sqrt{\frac{1}{n}\sum_{i=1}^{n}{\left({\mathrm{RD}}_{\mathrm{ij}}\right)}^{2}}\;$$

where $\mathrm{RMS}E_{j}$ is the RMSE at time point j. The time point with the smallest MAPE and RMSE was defined as the optimal time point for performing STP dosimetry. Standard errors (SEs) for derived quantities were approximated by first‐order error propagation, as detailed in the Supplemental File, Part A: Derivation of the Uncertainties.

To contextualize the performance of Scenarios 1–4 against existing methods, the analysis was divided into two distinct evaluations, as depicted in Figure 2: (a) single‐cycle STP dosimetry and (b) multi‐cycle STP dosimetry.

**FIGURE 2:**
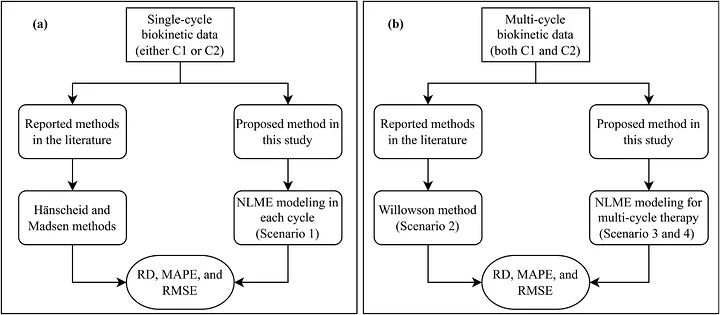
Schematic workflow for the comparison between the proposed single‐time‐point (STP) dosimetry methods and established methods in the literature. The figure distinguishes retrospective evaluation settings from the anticipated clinical information flow. All‐time‐point (ATP) data from other patients are used to define the population model, whereas STP or ATP data from the investigated patient represent the information available for individualized estimation under each scenario. **(a)** Single‐cycle STP dosimetry framework. Single‐cycle biokinetic data from either C1 or C2 were utilized to perform STP dosimetry within the respective cycle. The predictive performance of the proposed method (Scenario 1) was compared against the methods proposed by Hänscheid et al.^8^ and Madsen et al.^9^ **(b)** Multi‐cycle STP dosimetry evaluation. In this framework, biokinetic data from both C1 (using either ATP or STP data) and C2 were incorporated to evaluate STP dosimetry in C2. The proposed cross‐cycle methods (Scenarios 3 and 4) were compared against the approach described by Willowson et al.^15^ (Scenario 2). Final predictive accuracy across all frameworks was assessed using relative deviation (RD), mean absolute percentage error (MAPE), and root‐mean‐square error (RMSE).

In the single‐cycle STP dosimetry framework, single‐cycle biokinetic data from either C1 or C2 were used. The predictive performance of the proposed method (Scenario 1) was compared against reported methods in the literature, specifically those proposed by Hänscheid et al.^8^ and Madsen et al.^9^ For these comparisons, the sTIACs for the Hänscheid and Madsen methods were approximated using Equation (9) and Equation (10), respectively:

(9) $$\mathrm{sTIA}C_{H}=A\left(t_{\mathrm{sc}}\right)\times \frac{2\times t_{\mathrm{sc}}}{\ln2}$$

(10) $$\mathrm{sTIA}C_{M}=\frac{A\left(t_{\mathrm{sc}}\right)e^{\hat{k}t_{\mathrm{sc}}}}{\hat{k}}\;$$

where $t_{sc}$ is the SPECT/CT imaging time point after injection, $A(t_{sc})$ represents the fractional activity at time $t_{sc}$, and $\hat{k}$ denotes the population effective clearance rate for the monoexponential function.

In contrast, the multi‐cycle STP dosimetry framework utilized biokinetic data from multiple cycles (both C1 and C2). Our proposed cross‐cycle methods (Scenarios 3 and 4) were compared against the reported method in the literature, namely the approach described by Willowson et al.^15^ (Scenario 2). Through this multi‐cycle approach, we investigated the integration of multi‐cycle data to improve ongoing therapeutic dosimetry. Specifically, within our dataset, we incorporated C1 data (comprising either STP or ATP imaging) to enhance the predictive performance of STP dosimetry in C2. Scenarios 1, 3, and 4 were evaluated retrospectively using leave‐one‐out cross‐validation to quantify how accurately sTIACs reproduced rTIACs. These scenarios should therefore be interpreted as evaluation settings rather than as fully prospective clinical workflows. In prospective use, the appropriate scenario would depend on whether prior‐cycle STP or ATP data are available for the individual patient and whether a validated population model is already established.

## 3 RESULTS

### 3.1 Reference model

Figure 3 presents the reference biokinetic curves for the left kidneys in ATP fitting in C1 and C2, incorporating all variability components defined in Equation (2). The corresponding curves for the right kidneys, including a direct comparison between the left and right kidneys, are presented in the Supplemental File (Figures S1–S3). Overall, the biokinetic curves were nearly identical between the left and right kidneys across the cohort; a slight difference was observed only for patients 1 and 2. All biokinetic curves met the goodness‐of‐fit criteria, demonstrating the ability of the model to fit the measured biokinetic data. Table 1 presents the estimated parameters of the function f(t) for both fixed and random effects, along with the respective RSEs. Additionally, the NLME modeling diagnostic plots are presented in the Supplemental File (Figure S4).

**FIGURE 3:**
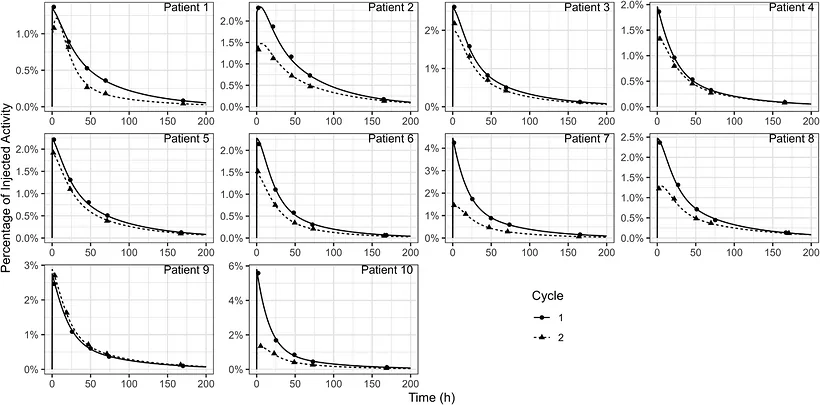
Reference biokinetic curves for the left kidneys with all‐time‐point (ATP) fitting considering the variability defined in Equation (2).

**TABLE 1:** The estimated parameters of the function f(t) (Equation (1)) from the multi‐cycle dataset using all‐time‐point (ATP) fitting.

Parameters (unit)	Fixed effects (%RSE)	IIV + Inter‐cycle variability^$^ (%RSE)	Kidney variability (%RSE)
A_1_ (%)	2.93 (15)	0.28 (20)	—^*^
A_2_ (%)	0.94 (10)	0.41 (17)	0.10 (24)
A_3_ (%)	1.83 (22)	—^*^	—^*^
$\lambda_{1\;}$(h^−1^)	6.64 × 10^−2^ (7)	—^*^	—^*^
$\lambda_{2\;}$(h^−1^)	9.30 × 10^−3^ (4)	0.12 (21)	—^*^
$\lambda_{3\;}$(h^−1^)	1.09 × 10^−1^ (11)	0.21 (41)	—^*^
Residual errors			
$\varepsilon_{1}$ on the first cycle		4.53 × 10^−2^ (12)	
$\varepsilon_{2}$ on the second cycle		6.44 × 10^−2^ (12)	

*Note*: The random effects are presented as a coefficient of variation (CV). The values in parentheses correspond to the relative standard errors (RSEs) of the estimated parameters.

* The random effects on the corresponding parameter were fixed to zero (i.e., no random effects) as the first minimization yielded a variance < 10^−6^.^34^

^$^ In the initial model, IIV and inter‐cycle variability were specified as separate random‐effect components as defined in Equation (2). Because the separately estimated IIV component was negligible/unstable in this small cohort, the final model used one combined random‐effect term varying across individuals and cycles. This term therefore reflects combined inter‐individual/inter‐cycle variability and should not be interpreted as a separately identifiable inter‐cycle effect.

### 3.2 Single‐time‐point dosimetry scenarios

Scenario 1: STP dosimetry using single‐cycle data yielded the lowest MAPE and RMSE in C1 at TP2 (23 ± 2) h after injection, which were (5.7 ± 1.7)% and (7.3 ± 2.0)%, respectively. In C2, TP4 (71 ± 2) h after injection was identified as the time point with the lowest MAPE and RMSE, which were (5.5 ± 1.9)% and (7.2 ± 2.6)%, respectively. The numerically optimal time point differed between C1 and C2; however, the Wilcoxon paired test did not show statistically significant differences between TP2 (23 ± 2) h and TP3 (47 ± 2) h in C1, or among TP2 (23 ± 2) h, TP3 (47 ± 2) h, and TP4 (71 ± 2) h in C2. The MAPE and RMSE for all time points in this scenario are reported in the Supplemental File, Table S1.

Scenario 2: The performance evaluation indicated that TP2 (23 ± 2) h in C2 yielded the lowest deviation, achieving a MAPE of 16.9% and an RMSE of 18.1%. In addition, a notably large error was observed at a late time point, with the MAPE reaching 770% and the RMSE reaching 1673%. Comprehensive values for the MAPE and RMSE in this scenario are reported in Table S2 of the Supplemental File, and the RD distributions are provided in Figure S5 of the Supplemental File.

Scenario 3: STP dosimetry using C1 and C2 time‐point combinations was investigated. The optimal combination, which minimized both MAPE and RMSE, was TP2 (23 ± 2) h after injection in C1 and TP4 (71 ± 2) h after injection in C2. For this optimal pairing, the MAPE and RMSE values were (4.5 ± 1.7)% and (5.7 ± 2.9)% in C1, and (6.5 ± 3.4)% and (7.4 ± 6.9)% in C2, respectively. Overall, the performance evaluation of Scenario 3 consistently yielded lower MAPE and RMSE values across most combinations (Supplemental File, Table S3) compared to Scenario 2 (Supplemental File, Table S2). Time‐point combinations involving TP2 (23 ± 2) h, TP3 (47 ± 2) h, and TP4 (71 ± 2) h in C1 resulted in the largest proportion of RDs ≤ 10% across the corresponding C2 time points (Figure 4). Although extreme RD values of up to 50% were observed for some combinations, and two specific cross‐cycle combinations (TP1 (2.5 ± 0.9) h in C1 paired with TP3 (47 ± 2) h in C2 and TP1 (2.5 ± 0.9) h in C1 paired with TP4 (71 ± 2) h in C2) resulted in two extreme RDs reaching up to 109% for one patient, both MAPE and RMSE improved consistently relative to Scenario 2. Comprehensive MAPE and RMSE values for this scenario are detailed in Table S3 of the Supplemental File.

**FIGURE 4:**
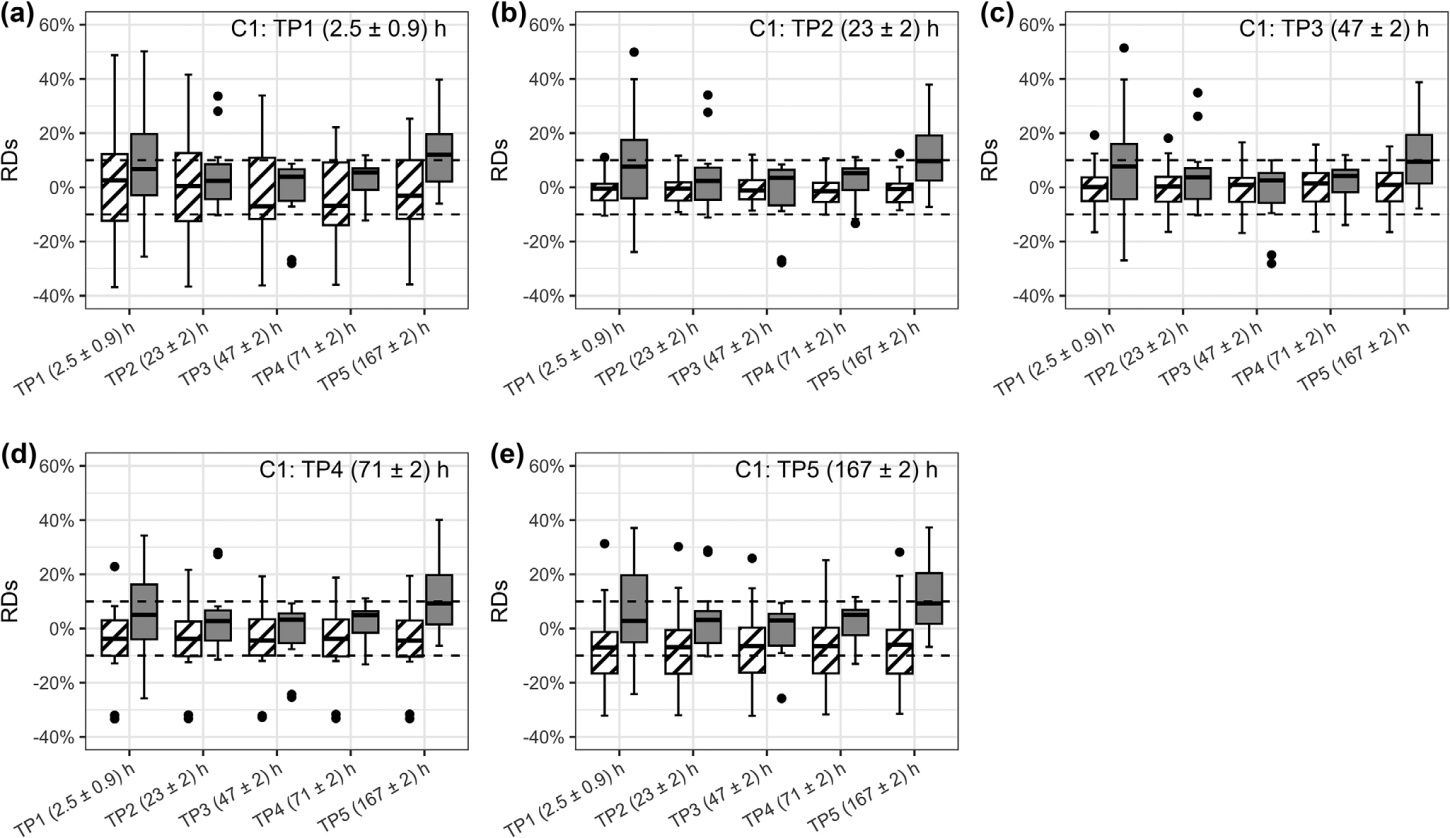
Relative deviations (RDs) of time‐integrated activity coefficients (TIACs) obtained from Scenario 3: single‐time‐point (STP) fitting using all combinations of time points between the first cycle (C1) and the second cycle (C2). In each panel, the C1 time point is fixed, while the x‐axis denotes the corresponding C2 time point. White boxplots with black diagonal stripes represent RDs for C1, and solid gray boxplots represent RDs for C2. Horizontal dashed lines indicate an absolute RD of 10%. Panels (a)–(e) correspond to STP fitting using TP1 (2.5 ± 0.9) h—TP5 (167 ± 2) h in C1, respectively, each combined with TP1 (2.5 ± 0.9) h—TP5 (167 ± 2) h in C2. **(a)** C1: TP1 (2.5 ± 0.9) h combined with all C2 time points; two kidney datasets (one patient) for TP3 (47 ± 2) h in C2, and two kidney datasets (one patient) for TP4 (71 ± 2) h in C2 were not shown due to extreme RDs (RD up to 109%). **(b)** C1: TP2 (23 ± 2) h combined with all C2 time points. **(c)** C1: TP3 (47 ± 2) h combined with all C2 time points. **(d)** C1: TP4 (71 ± 2) h combined with all C2 time points. **(e)** C1: TP5 (167 ± 2) h combined with all C2 time points.

Scenario 4 used ATP dosimetry in C1 and STP dosimetry in C2, resulting in improvements in both MAPE and RMSE, particularly at the early time points. The optimal time points for performing STP in C2 in this scenario were TP2 (23 ± 2) h, TP3 (47 ± 2) h, and TP4 (71 ± 2) h after injection. At these optimal time points, the observed MAPEs were (7.8 ± 2.4)%, (7.8 ± 2.6)%, and (6.7 ± 3.4)% for TP2 (23 ± 2) h, TP3 (47 ± 2) h, and TP4 (71 ± 2) h, respectively, while the RMSE values were (11.1 ± 2.0)%, (10.7 ± 3.4)%, and (7.5 ± 7.0)%, respectively. The early time point demonstrated a MAPE of (13.9 ± 5.0)%, indicating relatively good agreement between the sTIACs and rTIACs for this scenario. The complete MAPE and RMSE values for Scenario 4 are provided in Table S4, and RDs across time points are presented in Figure S6 in the Supplemental File.

### 3.3 Single‐time‐point dosimetry for single‐cycle and multi‐cycle framework

The proposed single‐cycle NLME method, Scenario 1, consistently maintained optimal MAPEs below 6% (as detailed previously); the method reported in the literature yielded higher RDs. Specifically, the Hänscheid method yielded optimal MAPEs of 13% and 16% for C1 and C2, respectively, occurring at TP3 (47 ± 2) h after injection for both cycles. The Madsen method achieved optimal MAPEs of 5.3% and 10.7% for C1 and C2, respectively, both occurring at TP2 (23 ± 2) h after injection. Figure 5 demonstrates a systematic bias within both the Hänscheid and Madsen methods. The Hänscheid method consistently underestimated the rTIACs, observed from TP2 (23 ± 2) h to TP4 (71 ± 2) h after injection across both cycles. Conversely, the Madsen method exhibited a time‐dependent bias across both cycles: it tended to overestimate the rTIACs at the early and late time points, while underestimating them at the intermediate time points from TP2 (23 ± 2) h to TP4 (71 ± 2) h after injection. Comprehensive MAPE and RMSE values for the Hänscheid and Madsen methods are provided in the Supplemental File, Tables S5,S6.

**FIGURE 5:**
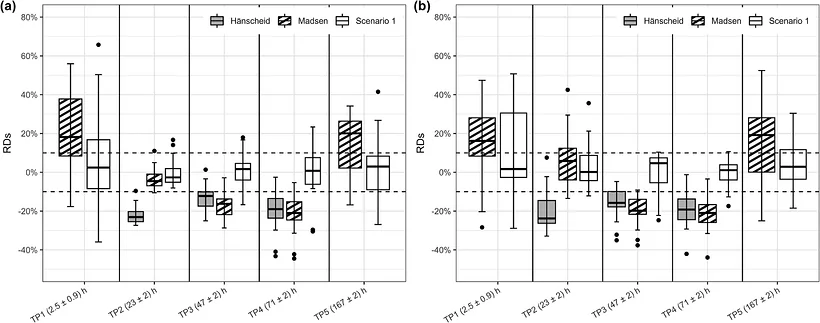
Relative deviations (RDs) of time‐integrated activity coefficients (TIACs) derived from single‐time‐point (STP) dosimetry within single‐cycle frameworks. Horizontal dashed lines represent an absolute RD of 10%. Evaluations at TP1 (2.5 ± 0.9) h and TP5 (167 ± 2) h for the Hänscheid method were omitted because these time points fall outside the effective validity interval for this method.^8^ **(a)** Evaluation in the first cycle (C1). Two data points from the Madsen method at TP1 (2.5 ± 0.9) h are not shown due to extreme RD values (∼101%). **(b)** Evaluation in the second cycle (C2). Two data points from the Madsen method at TP5 (167 ± 2) h are not shown due to extreme RD values (∼90%).

Within the multi‐cycle evaluation, Scenario 2 (the STP method reported in the literature) exhibited the highest overall RDs compared to Scenarios 3 and 4. In comparison, the proposed multi‐cycle NLME frameworks (Scenarios 3 and 4) maintained higher accuracy, with multiple time‐point combinations resulting in MAPEs below 10%. To provide a clear comparative overview, Figure 6 summarizes the predictive performance across these multi‐cycle STP dosimetry methods, specifically at the early time point TP1 (2.5 ± 0.9) h after injection during C2. This focus on the early C2 time point reflects the primary clinical objective of STP dosimetry: utilizing comprehensive or STP biokinetic data established in C1 to reliably streamline and predict dosimetry at an early time point in subsequent cycles.

**FIGURE 6:**
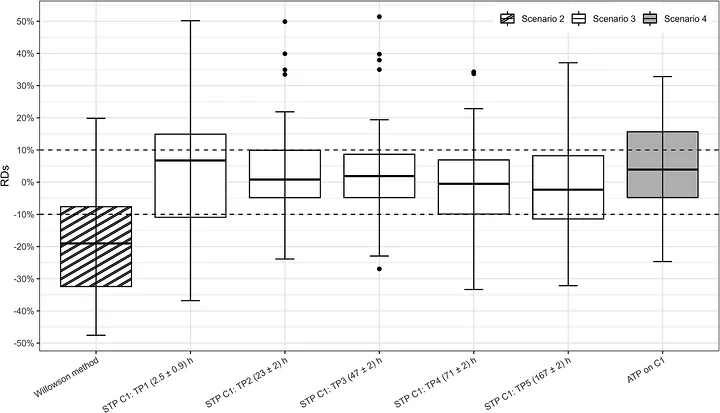
Relative deviations (RDs) of time‐integrated activity coefficients (TIACs) derived from single‐time‐point (STP) dosimetry evaluated at TP1 (2.5 ± 0.9) h after injection during the second cycle (C2), compared across multi‐cycle therapy scenarios. Horizontal dashed lines represent absolute RDs of 10%. The evaluated methods include one method reported in the literature alongside two methods proposed in this study. Scenario 2 represents the existing cross‐cycle method utilizing a monoexponential fit function, as reported by Willowson et al.^15^ The proposed methods comprise Scenario 3, which utilizes STP dosimetry in both C1 and C2, and Scenario 4, which relies on ATP biokinetic data in C1 to improve the STP dosimetry in C2.

## 4 DISCUSSION

### 4.1 Parameter interpretation

In this study, we employed the fit function from the previous study^13^ (Equation 1). We fitted our multi‐cycle data using a modified statistical model (Equation 2) and demonstrated that the NLME model adequately described the biokinetic data. The $A_{j}\left(j=1,2,3\right)$ parameter distributions related to the fixed and random effects in this study differ from those in a prior study at a different institution investigating a different patient population.^13^ This divergence is likely due to differences in administered activity, as well as the distinct diseases of the respective cohorts. Specifically, the present study utilized a population with low‐volume mHSPC receiving two therapy cycles with varying administered activities (median administered activity of 3 GBq in C1 and 6 GBq in C2). In contrast, the previous study^13^ included a single‐cycle dataset from patients with mCRPC who received a median administered activity of 6 GBq. While this difference in disease state and administered activity may limit the direct generalizability of the $A_{j}\left(j=1,2,3\right)$ parameters across all prostate cancer populations, the $\lambda_{j}\left(j=1,2,3\right)$ parameters are similar between the two studies, indicating that renal uptake and clearance of [^177^Lu]Lu‐PSMA‐617 are consistent across different centers and patient populations. However, baseline renal‐function markers were not included as covariates in the present NLME analysis. Consequently, potential effects of individual renal function on the uptake or clearance parameters cannot be definitively excluded. While investigating the direct influence of these clinical biomarkers is beyond the scope of the current study, this should be assessed in future investigations utilizing larger cohorts and predefined covariate‐modeling strategies.

The statistical models in this study included three variability components (Equation 2) and two residual‐variability terms (Equation 4), with one residual variability for each cycle. The fitting results indicated that inter‐cycle variability is larger than IIV (data not shown), which may reflect the limited sample size (10 patients) or a characteristic cycle‐to‐cycle trend. Validation using a larger population size is required to determine whether this pattern persists and whether IIV and inter‐cycle variability can be reliably estimated separately. In the current study, IIV and inter‐cycle variability were combined because separate estimation yielded large RSEs (RSEs > 50%) or negligible IIV estimates (i.e., variance $<10^{-6}$). When merged, the parameters are estimated precisely (Table 1), indicating that, for our dataset, the combined variability provides a more stable model. In addition to IIV and inter‐cycle variability, our results (Table 1) also report the variability of the $A_{2}$ parameter between the left and right kidneys. When interpreting these estimates, it must be noted that paired kidneys from the same patient are not statistically independent. Although the estimated kidney variability for the parameter $A_{2}$ was classified as small (10% CV) based on the criteria reported in the literature,^26^, ^37^ the variability for parameter $A_{2}$ was retained in the final model due to its precise estimation (RSE of 24%). This parameter ensures the model accurately captures the slight differences in the left‐right biokinetic curves observed in a subset of patients (Supplemental File, Figures S2 and S3) rather than inflating the residual error.^25^, ^26^, ^38^ Finally, the residual variability between the two cycles was similar, suggesting comparable model performance across both cycles.

### 4.2 Single‐time‐point dosimetry scenarios

Because the rTIACs were derived from ATP fitting rather than from an independent ground truth, the reported STP performance reflects agreement with a model‐based reference. External validation in independent cohorts and sensitivity analyses using alternative reference fitting approaches would strengthen the evidence for clinical generalizability. Clinically, while the shift in the optimal STP dosimetry time point from TP2 (23 ± 2) h in C1 to TP4 (71 ± 2) h in C2 was unexpected, the lack of statistical difference indicates that STP dosimetry can be performed at either TP2 (23 ± 2) h or TP3 (47 ± 2) h for both cycles. However, interpreting the clinical acceptability of this flexibility is complicated because of the lack of consensus regarding an acceptable error threshold in clinical dosimetry. As a result, defining a specific predictive error as “viable” remains highly subjective. For instance, Gomes et al.^39^ stated that the early time point yielding a MAPE of 27% provided viable results for STP dosimetry; in contrast, if a stricter clinical threshold of 20% were applied, those same results would be deemed unacceptable. Furthermore, although the observed shift in the numerically optimal time point from TP2 (23 ± 2) h in C1 to TP4 (71 ± 2) h in C2 could suggest subtle cycle‐dependent kinetic differences, the absence of statistically significant differences among neighboring time points and the small cohort size preclude a definitive interpretation. Nevertheless, these findings align with Peters et al.,^10^ who, despite using different STP methods, also reported that STP fitting for normal tissues (e.g., kidneys) can be performed at TP2 (23 ± 2) h or TP3 (47 ± 2) h after injection. This consistency regarding the optimal time point is further supported by our comparative analysis: regardless of the overall accuracy of the respective methods, the Madsen^9^ and Hänscheid^8^ methods yielded optimal time points at TP2 (23 ± 2) h and TP3 (47 ± 2) h, respectively. Consequently, across multiple distinct methods evaluated within a single‐cycle framework in our dataset, the optimal time point for performing STP dosimetry remains robustly at either TP2 (23 ± 2) h or TP3 (47 ± 2) h. Prior results^13^, ^40^ further reported TP3 (47 ± 2) h as the optimal time point based on the lowest MAPE and RMSE values, reinforcing the present finding when using the single‐cycle dataset.

Previous studies^13^, ^40^ have highlighted that a monoexponential fit function in STP dosimetry, such as those employed in the Hänscheid et al.^8^ and Madsen et al.^9^ methods, may introduce bias when compared to STP dosimetry using either NLME modeling or a Bayesian method with a fit function selected through the model selection.^17^, ^18^ Our findings align with these previous observations.^13^, ^40^ As detailed in our single‐cycle evaluation, the reliance on this frequently used monoexponential function resulted in systematic underestimations and time‐dependent biases across both cycles. The biases observed are likely due to monoexponential biokinetic assumptions, which may not adequately capture the multi‐phase biokinetic profile of the radiopharmaceutical. In contrast, such systematic bias was not observed in our proposed NLME modeling framework (Scenarios 1, 3, and 4), which naturally accommodates multi‐phase biological clearance in the kidneys as depicted in Equation (1). These discrepancies underscore the critical importance of selecting an appropriate structural fit function for accurate dosimetry. Therefore, the model selection method^17^, ^18^ is recommended to identify the fit function most supported by the data and may enhance the reproducibility of fit‐function selection.^17^, ^18^, ^21^ If model selection is not performed, it is advisable to use the fit function that has already been shown to be most supported by the data, provided that the same radiopharmaceutical is used. Related PBMS NLME modeling approaches have also been evaluated outside PSMA therapy, including radioiodine therapy,^41^ suggesting broader methodological applicability while reinforcing that radiopharmaceutical‐, organ‐, and disease‐specific structural models remain necessary.

As demonstrated in Figure S5 of the Supplemental File, the sTIACs at TP1 (2.5 ± 0.9) h to TP3 (47 ± 2) h derived from Scenario 2 tend to underestimate the expected values (i.e., rTIACs) and introduce a large deviation at TP4 (71 ± 2) h and TP5 (167 ± 2) h. This bias may arise due to the assumption that the effective half‐life remains constant at the value estimated from the ATP fitting in C1. This assumption may lead to an inaccurate effective half‐life value in C2 and, consequently, introduce a bias in the calculation of the A value in the monoexponential function, thereby causing a systematic deviation in the TIAC estimation. Extreme RDs using this method were observed at the late time point TP5 (167 ± 2) h after injection, with MAPE and RMSE values of 770% and 1673%, respectively. The very large TP5 (167 ± 2) h deviations should primarily be interpreted as evidence of extrapolation failure at late, low‐activity measurements, rather than as the central basis for comparing methods. The previous report by Willowson et al.^15^ did not capture this finding, possibly because their dataset only extended up to 120 h after injection. While late time points (up to one week after injection) may not typically be clinically relevant times for performing STP dosimetry in [^177^Lu]Lu‐PSMA‐617 therapy, this finding remains informative. It suggests that late time points are outside the intended window of STP methods such as the method proposed by Hänscheid et al.^8^

Clinically, implementing Scenario 3 requires the initial STP dosimetry time points in C1. This requirement arises because the direct application of STP fitting to both C1 and C2 simultaneously is impractical in clinical routine. Because data are obtained first in C1 and then in C2, C1 time points can be selected according to Scenario 3, employing only C1 data for the specific patient under investigation and the entire dataset (C1 and C2) for the remaining patients. The result of STP fitting in C1 without any C2 data in Scenario 3 is comparable to the C1‐specific results detailed in Supplemental File, Table S3. This similarity underscores that Scenario 3 can be conducted by first performing the procedure in C1 and subsequently combining the results with STP data for C2, while maintaining similar levels of accuracy.

The overall performance improvements observed in Scenario 3 indicate that multi‐cycle STP fitting offers greater flexibility in time‐point selection. These findings suggest that incorporating inter‐cycle variability may improve the predictive performance of rTIACs derived from STP biokinetic data in each cycle. Accordingly, the inclusion of inter‐cycle variability within multi‐cycle STP dosimetry using NLME modeling is recommended. Furthermore, despite achieving lower MAPE values than those reported by Gomes et al.^39^ for early time points, the results underscore that STP fitting performed at suboptimal time points (e.g., at TP1 (2.5 ± 0.9) h) should be applied with caution, as it may lead to substantial individual‐level errors in some patients. Nevertheless, the overall set of time‐point combinations investigated in our study yielded lower MAPEs and fewer outliers than those reported by Gomes et al.^39^ These findings support further evaluation of NLME‐based STP dosimetry in multi‐cycle RPT.

The application of ATP biokinetic data from C1 combined with STP biokinetic data from C2 in Scenario 4 resulted in improved accuracy for sTIAC estimation within C2 (Supplemental File, Table S4) compared to the results of Scenario 2 (Supplemental File, Table S2). Specifically, the incorporation of ATP biokinetic data in C1 appeared to mitigate the occurrence of outliers, as evidenced by notably lower RMSEs than in Scenario 2. Furthermore, the MAPE at early time points for Scenario 4 improved, suggesting the feasibility of STP dosimetry at these time points. This improved performance (MAPE ∼14%) contrasts favorably with the literature; for instance, the method proposed by Gomes et al.,^39^ which reported a ∼27% MAPE at early time points, was suggested as a viable approach for flexible STP dosimetry. Our findings for Scenario 4, indicated by lower MAPE and RMSE values, reflect a better agreement between sTIACs and rTIACs. Consequently, Scenario 4 appears to offer greater flexibility for STP dosimetry during the second cycle and possibly subsequent cycles. A key drawback in Scenario 4, however, is the requirement for five time points in C1, which could increase the patient burden and clinical workload. As an alternative, a different scenario may be selected for early‐time‐point STP dosimetry, with the C2 performance of early‐time‐point STP dosimetry illustrated in Figure 6.

The TIACs in ATP fitting showed good agreement in both TIAC values and their uncertainties between the single‐cycle and multi‐cycle datasets (RDs ≤ 2%). These results may indicate that, when ATP fitting (i.e., five time points) is used, the inclusion of multi‐cycle data does not substantially change either the TIAC estimates or their precision. In contrast, a noticeably lower precision was observed in STP fitting. The present findings are also consistent with previous accuracy and precision analyses of STP dosimetry using PBPK and NLME modeling, which highlighted that sparse sampling can be clinically attractive but requires explicit evaluation of uncertainty and protocol robustness.^42^ The broader uncertainty of the performance metrics observed in Scenario 3 may reflect the additional variability introduced when multi‐cycle information is modeled with a combined inter‐individual and inter‐cycle random‐effect structure. Because full covariance propagation was not performed, this interpretation should be considered qualitative. Nevertheless, the corresponding loss of precision was small relative to the improvement in agreement with the ATP‐derived model‐based reference TIACs.

### 4.3 Clinical application and limitations

To facilitate the translation of the proposed multi‐cycle STP dosimetry method in this study into routine clinical practice, we propose the following structured, stepwise workflow for implementing NLME‐based STP dosimetry:

1. Population Data Acquisition: The prerequisite for NLME modeling is a prior population dataset. Clinics can utilize local historical data, collect data from the literature, or establish multi‐center data‐sharing agreements. However, multi‐center data‐sharing may require standardized imaging and quantification protocols to ensure quantitative data uniformity.
2. Model Development and Validation: Using a representative population dataset, the parameters of the structural model (e.g., Equation (1)) and the statistical model (e.g., Equation (2)) are estimated within the NLME modeling framework. The model must then satisfy the predefined goodness‐of‐fit criteria to ensure parameter identifiability and its ability to describe the biokinetic data in ATP fitting. In the context of STP dosimetry, the model should be considered suitable for a specific clinical workflow only if cross‐validation and, ideally, external validation demonstrate that prespecified, clinically justified accuracy and precision criteria are met. To validate the accuracy of the model for STP dosimetry, leave‐one‐out cross‐validation can be performed using the available cohort data. If the objective is to validate the C2 STP dosimetry by using the C2 STP measurement together with ATP biokinetic data from other patients, while incorporating C1 STP biokinetic data as prior patient‐specific information, then Scenario 3 represents the appropriate validation setting. In contrast, if the intended clinical workflow uses ATP biokinetic data from C1 to support STP dosimetry validation in C2, then Scenario 4 is the more suitable option. Ideally, this validation should first be performed using the clinic's internal dataset and subsequently confirmed in an independent external cohort, if available. This implementation pathway is consistent with recent reviews of population pharmacokinetics with NLME modeling in radiopharmaceutical therapy, which emphasize that sparse‐data dosimetry requires representative population data, harmonized quantitative imaging, transparent model diagnostics, and external validation before routine clinical adoption.^43^
3. STP Fitting for a New Patient: When STP biokinetic data are available for a new patient in C1 or C2, patient‐specific pharmacokinetic parameters can be estimated from the single observation by fitting the patient's STP measurement jointly with the available population dataset within the NLME framework. The appropriate scenario should be selected according to the intended clinical application. For STP dosimetry confined to a single‐cycle therapy, Scenario 1 may be sufficient. In contrast, when the aim is to support multi‐cycle STP dosimetry by incorporating prior‐cycle patient‐specific information or cross‐cycle population data, Scenario 3 or Scenario 4 should be used, depending on the validation or clinical‐use setting described above.
4. Clinical Evaluation and Interpretation: Although the overall predictive performance of the model is assessed through its predictive accuracy, application to an individual patient requires careful consideration of the uncertainty associated with the patient‐specific estimate. Therefore, the derived dosimetric quantities should be interpreted together with their corresponding uncertainty estimates, such as confidence intervals, as estimated within the NLME framework. Incorporating these uncertainty estimates during treatment planning and response evaluation provides a quantitative measure of the precision of individual dosimetry results and supports informed clinical interpretation of the estimated absorbed dose.

A limitation of the present study is that the utilized structural model (Equation (1)) is specifically tailored for renal dosimetry in [^177^Lu]Lu‐PSMA‐617 therapy. Consequently, this specific SOEF cannot be directly employed for tumors or other organs at risk, such as the salivary glands, lacrimal glands, or bone marrow, which may exhibit different pharmacokinetic behaviors. For the dosimetric evaluation of these tissues, an appropriate structural model must first be selected. This can be achieved either by adopting previously published fit functions specific to those tissues (e.g., established models for tumor dosimetry in [^177^Lu]Lu‐PSMA‐617)^16^ or by performing model selection to identify the function most supported by the organ dataset.^17^, ^18^ Once a suitable organ‐specific structural model is selected, the STP dosimetry workflow presented in this study (Figure 2) can be readily adapted.

This study is limited by its small cohort (10 patients, 20 kidneys, and 40 kidney‐cycle datasets) and by its restriction to the first two therapy cycles. Although the fitted clearance‐rate parameters did not indicate a large change between C1 and C2, it remains unknown whether this stability persists during standard six‐cycle regimens or after repeated administrations of approximately 7.4 GBq. Later‐cycle changes in renal function, tumor burden, hydration status, concomitant medication, or systemic pharmacokinetics could alter the applicability of the current model. Furthermore, with more data, it is expected that all variability components defined in Equation (2) could be estimated separately rather than being merged (Table 1). Although using different radiopharmaceuticals and structural models, Areberg et al.^44^ demonstrated that inter‐cycle variability and IIV can indeed be estimated separately within large populations. Nevertheless, despite the small dataset, the present study remains highly informative. The model met the goodness‐of‐fit criteria, and the results successfully demonstrated the impact of incorporating multi‐cycle data, managing inter‐cycle variability within the NLME modeling framework, and optimizing STP time‐point combinations across different cycles.

## 5 CONCLUSION

This study demonstrates that incorporating multi‐cycle biokinetic data into STP dosimetry within the NLME modeling framework improves agreement between sTIACs and rTIACs for renal dosimetry in two cycles of [^177^Lu]Lu‐PSMA‐617 therapy. The integration of multi‐cycle data offers greater flexibility in determining suitable time points for STP dosimetry compared to analyses that are limited to single‐cycle data. Larger external cohorts including later treatment cycles are required to establish whether this method can be generalized to routine multi‐cycle PSMA therapy.

## CONFLICT OF INTEREST STATEMENT

Deni Hardiansyah and Gerhard Glatting are independent consultants for ITM Oncologics GmbH. The authors have no other relevant conflicts of interest to disclose.

## Supporting information




Supporting Information
