# Supplementary material for: Single‐time‐point renal dosimetry for [177Lu]Lu‐PSMA‐617: Incorporating multi‐cycle population data using nonlinear mixed‐effects models
Source: Med Phys. 2026 Oct 22;53(11):e70736. doi: 10.1002/mp.70736 (PMC13691518; doi:10.1002/mp.70736)
Supplement: Supplementary file 1 [file MP-53-0-s001.pdf]

Supplemental File

Single-Time-Point Renal Dosimetry for [^{177}Lu]Lu-PSMA-617: Incorporating Multi-Cycle Population Data Using Nonlinear Mixed-Effects Models

Indra Budiansah[1,2], Steffie M. B. Peters[3], Bastiaan Privé[3,4], James Nagarajah[3,5], Mark W. Konijnenberg[3,6], Gerhard Glatting[2$], Deni Hardiansyah[1,2*$]

[1]*Medical Physics and Biophysics Division, Physics Department, Faculty of Mathematics and Natural Sciences, Universitas Indonesia, Depok, 16424, Indonesia*

[2]*Medical Radiation Physics, Department of Nuclear Medicine, Ulm University, Ulm, Germany*

[3]*Department of Medical Imaging, Radboud University Medical Center, 9101, 6500 HB Nijmegen, The Netherlands*

[4]*Department of Radiology and Nuclear Medicine, Erasmus Medical Center, Rotterdam, The Netherlands*

[5]*Röntgeninstitut Düsseldorf, Düsseldorf, Germany*

[6]*Department of Radiation Oncology, Erasmus Medical Center, Rotterdam, The Netherlands*

[$]shared senior authorship
*Corresponding Author:
Dr. sc. hum. Deni Hardiansyah
Physics Department, Faculty of Mathematics and Natural Sciences (FMIPA),
Universitas Indonesia, Depok, 16424
Email (University): denihardiansyah@ui.ac.id

Part A: Derivation of the Uncertainties

The derivation is based on the literature.[1] This propagation assumes independence between the individual RD values and does not include covariance terms between sTIAC and rTIAC estimates. In the leave-one-out NLME framework, this independence assumption is approximate because estimates are derived from related structural models and partially overlapping population information. Given that sTIACs and rTIACs were derived from related model fits and that measurements were clustered within patients, these propagated SEs should be interpreted as approximate descriptive precision estimates rather than as full uncertainty quantification. Because two kidneys and repeated therapy cycles were available per patient, the performance metrics were calculated at the kidney-dataset level but interpreted as clustered descriptive measures rather than as fully independent observations. Formal statistical comparisons between time points were therefore considered exploratory.

Relative Deviations (RDs)

Suppose the obtained sTIAC and rTIAC are $(a \pm \Delta a)$ and $(b \pm \Delta b)$, respectively. The RDs were calculated using Eq. (6) of the main manuscript. The uncertainty of RD can be obtained as follows:

$$\begin{aligned}
\Delta\mathrm{RD} &= \sqrt{\left(\frac{\partial \mathrm{RD}}{\partial a}\mathrm{d}a\right)^2 + \left(\frac{\partial \mathrm{RD}}{\partial b}\mathrm{d}b\right)^2} \\
\Delta\mathrm{RD} &= \sqrt{\left(\frac{\mathrm{d}a}{b}\right)^2 + \left(\frac{-b\cdot \mathrm{d}b - (a-b)\mathrm{d}b}{b^2}\right)^2} \\
\Delta\mathrm{RD} &= \sqrt{\left(\frac{\mathrm{d}a}{b}\right)^2 + \left(\frac{-a\cdot \mathrm{d}b}{b^2}\right)^2} \\
\Delta\mathrm{RD} &= \sqrt{\left(\frac{\Delta a}{b}\right)^2 + \left(\frac{a\Delta b}{b^2}\right)^2}
\end{aligned} \tag{S1}$$

Mean and Standard Deviation Uncertainties

The mean RD m and its uncertainty Δm can be calculated as follows:

$$m = \sum_{i=1}^{n} \frac{\mathrm{RD}_i}{n} \tag{S2}$$

with i being the index of the kidney dataset and n the total number of kidney datasets. Δm can be calculated as:

$$\begin{aligned}
\Delta m &= \sqrt{\sum_{i=1}^{n}\left(\frac{\partial m}{\partial \mathrm{RD}_i}\,\mathrm{d}\,\mathrm{RD}_i\right)^2} \\
\Delta m &= \sqrt{\sum_{i=1}^{n}\left(\frac{\partial}{\partial \mathrm{RD}_i}\left(\sum_{j=1}^{n}\frac{\mathrm{RD}_j}{n}\right)\mathrm{dRD}_i\right)^2} \\
\Delta m &= \sqrt{\sum_{i=1}^{n}\left(\sum_{j=1}^{n}\frac{\delta_{ij}\,\mathrm{dRD}_j}{n}\right)^2} \\
\Delta m &= \sqrt{\sum_{i=1}^{n}\left(\frac{\Delta \mathrm{RD}_i}{n}\right)^2}
\end{aligned} \tag{S3}$$

The sample standard deviation SD was calculated as

$$\mathrm{SD} = \sqrt{\sum_{i=1}^{n}\frac{(\mathrm{RD}_i - m)^2}{n-1}} \tag{S4}$$

where m is the mean of RD as defined in Eq. (S2). The uncertainty of SD can be calculated as:

$$\Delta \mathrm{SD} = \sqrt{\sum_{i=1}^{n}\left(\frac{\partial \mathrm{SD}}{\partial \mathrm{RD_i}}\,\mathrm{dRD_i}\right)^2} \tag{S5}$$

Calculating $\frac{\partial \mathrm{SD}}{\partial \mathrm{RD}_i}$ simplifies Eq. (S5):

$$\begin{aligned}
\frac{\partial \mathrm{SD}}{\partial \mathrm{RD}_i} &= \frac{\partial}{\partial \mathrm{RD}_i}\sqrt{\sum_{j=1}^{n}\frac{\left(\mathrm{RD}_j - m\right)^2}{n-1}} \\
&= \frac{1}{2\sqrt{\sum_{j=1}^{n}\frac{\left(\mathrm{RD}_j - m\right)^2}{n-1}}}\frac{\partial}{\partial \mathrm{RD}_i}\left[\sum_{j=1}^{n}\frac{\left(\mathrm{RD}_j - m\right)^2}{n-1}\right] \\
&= \frac{1}{2\cdot \mathrm{SD}}\sum_{j=1}^{n}\frac{1}{n-1}\cdot 2\left(\mathrm{RD}_j - m\right)\cdot\frac{\partial}{\partial \mathrm{RD}_i}\left(\mathrm{RD}_j - m\right) \\
&= \frac{1}{2\cdot \mathrm{SD}}\sum_{j=1}^{n}\frac{1}{n-1}\cdot 2\left(\mathrm{RD}_j - m\right)\cdot\left(\delta_{ij} - \frac{1}{n}\right) \\
&= \frac{1}{(n-1)\cdot \mathrm{SD}}\sum_{j=1}^{n}\left(\mathrm{RD}_j - m\right)\left(\delta_{ij} - \frac{1}{n}\right)
\end{aligned}$$

$$= \frac{1}{(n-1)\cdot \mathrm{SD}}\left(\left(\sum_{j=1}^{n} \mathrm{RD}_j \delta_{ij}\right) - \left(\frac{1}{n}\sum_{j=1}^{n} \mathrm{RD}_j\right) - m + \frac{1}{n}\sum_{j=1}^{n} m\right)$$

$$= \frac{\mathrm{RD}_i - m}{(n-1)\cdot \mathrm{SD}}$$

Therefore, the uncertainty for SD can be written as:

$$\Delta \mathrm{SD} = \sqrt{\sum_{i=1}^{n}\left[\frac{\mathrm{RD}_i - m}{(n-1)\cdot \mathrm{SD}} \cdot \Delta \mathrm{RD}_i\right]^2} \qquad (S6)$$

Mean Absolute Percentage Error (MAPE) Uncertainty

The MAPE was calculated utilizing Eq. (7) of the main manuscript, and its uncertainty was derived as follows:

$$\Delta \mathrm{MAPE} = \sqrt{\sum_{i=1}^{n}\left(\frac{\partial \mathrm{MAPE}}{\partial \mathrm{RD}_i}\,\mathrm{dRD}_i\right)^2}$$

$$\Delta \mathrm{MAPE} = \sqrt{\sum_{i=1}^{n}\left(\frac{\partial}{\partial \mathrm{RD}_i}\left(\frac{1}{n}\sum_{j=1}^{n}\left|\mathrm{RD}_j\right|\right)\mathrm{dRD}_i\right)^2} \qquad \text{(S7)}$$

$$\Delta \mathrm{MAPE} = \frac{1}{n}\sqrt{\sum_{i=1}^{n}(\Delta \mathrm{RD}_i)^2}$$

Root-Mean-Square Error (RMSE) Uncertainty

The RMSE was calculated based on Eq. (8) of the main manuscript. The uncertainty of the RMSE (ΔRMSE) was calculated as:

$$\Delta \mathrm{RMSE} = \sqrt{\sum_{i=1}^{n}\left(\frac{\partial \mathrm{RMSE}}{\partial \mathrm{RD}_i}\,\mathrm{dRD}_i\right)^2}$$

$$\Delta \mathrm{RMSE} = \sqrt{\sum_{i=1}^{n}\left(\frac{\partial}{\partial \mathrm{RD}_i}\left(\sqrt{\frac{1}{n}\sum_{j=1}^{n}\left(\mathrm{RD}_j\right)^2}\right)\mathrm{dRD}_i\right)^2} \qquad \text{(S8)}$$

$$\Delta \mathrm{RMSE} = \sqrt{\sum_{i=1}^{n}\left(\frac{\mathrm{RD}_i}{n\cdot \mathrm{RMSE}}\,\Delta \mathrm{RD}_i\right)^2}$$

Tables

Table S1. Performance metrics of Scenario 1. Mean absolute percentage error (MAPE) and root-mean-square error (RMSE) of single-time-point (STP) dosimetry at each time point using a single-cycle dataset. The model did not account for the inter-cycle variability.

Time Point	Cycle 1		Cycle 2	
	MAPE (%)	RMSE (%)	MAPE (%)	RMSE (%)
TP1 (2.5 ± 0.9) h	18.7 ± 3.8	24.9 ± 2.5	19.6 ± 4.9	25.2 ± 3.5
TP2 (23 ± 2) h	5.7 ± 1.7	7.3 ± 2.0	8.7 ± 2.3	12.0 ± 1.7
TP3 (47 ± 2) h	6.8 ± 2.2	9.0 ± 2.7	8.5 ± 1.9	10.7 ± 1.5
TP4 (71 ± 2) h	9.8 ± 3.2	13.2 ± 4.2	5.5 ± 1.9	7.2 ± 2.6
TP5 (167 ± 2) h	12.6 ± 3.8	16.8 ± 4.5	12.0 ± 3.1	15.9 ± 2.3

Table S2. Performance metrics of Scenario 2. The mean absolute percentage error (MAPE) and root-mean-square error (RMSE) of single-time-point (STP) in the second cycle (C2) were estimated using a monoexponential fit function with the effective half-life derived from all-time-point (ATP) fitting in the first cycle (C1) dataset, as reported in the literature.[2] The uncertainties of the MAPE and RMSE were not reported for this scenario because this approach replicated the existing method,[2] which did not account for the precision of these metrics.

Time Point	MAPE (%)	RMSE (%)
TP1 (2.5 ± 0.9) h	22.1	25.7
TP2 (23 ± 2) h	16.9	18.1
TP3 (47 ± 2) h	17.9	20.6
TP4 (71 ± 2) h	25.3	46.7
TP5 (167 ± 2) h	770	1673

Table S3. Performance metrics of Scenario 3. Mean absolute percentage error (MAPE) and root-mean-square error (RMSE) of single-time-point (STP) fitting for cycle 1 (C1) and cycle 2 (C2) in multi-cycle scenarios for all (full-grid) time-point combinations between C1 and C2.

Time Point in C1	Time Point in C2	First Cycle (C1)		Second Cycle (C2)	
		MAPE (%)	RMSE (%)	MAPE (%)	RMSE (%)
TP1 (2.5 ± 0.9) h	TP1 (2.5 ± 0.9) h	19.4 ± 4.9	24.2 ± 4.5	17.8 ± 5.4	23.8 ± 4.5
TP1 (2.5 ± 0.9) h	TP2 (23 ± 2) h	16.1 ± 4.6	19.6 ± 5.4	8.7 ± 2.5	12.1 ± 2.0
TP1 (2.5 ± 0.9) h	TP3 (47 ± 2) h	24.8 ± 4.9	39.2 ± 2.3	8.1 ± 2.6	11.1 ± 3.0
TP1 (2.5 ± 0.9) h	TP4 (71 ± 2) h	23.3 ± 4.7	37.9 ± 2.3	6.8 ± 3.2	7.6 ± 5.9
TP1 (2.5 ± 0.9) h	TP5 (167 ± 2) h	14.1 ± 4.5	17.2 ± 6.1	14.0 ± 4.6	18.4 ± 4.1
TP2 (23 ± 2) h	TP1 (2.5 ± 0.9) h	4.6 ± 1.8	5.8 ± 2.9	17.2 ± 5.3	22.1 ± 4.8
TP2 (23 ± 2) h	TP2 (23 ± 2) h	4.4 ± 1.8	5.7 ± 2.9	8.2 ± 2.5	11.7 ± 2.0
TP2 (23 ± 2) h	TP3 (47 ± 2) h	4.3 ± 1.8	5.4 ± 3.1	8.1 ± 2.6	11.1 ± 3.3
TP2 (23 ± 2) h	TP4 (71 ± 2) h	4.5 ± 1.7	5.7 ± 2.9	6.5 ± 3.4	7.4 ± 6.9
TP2 (23 ± 2) h	TP5 (167 ± 2) h	4.2 ± 1.7	5.3 ± 3.1	13.3 ± 4.5	17.7 ± 4.1
TP3 (47 ± 2) h	TP1 (2.5 ± 0.9) h	6.5 ± 1.9	8.9 ± 2.3	16.8 ± 5.3	22.4 ± 4.8
TP3 (47 ± 2) h	TP2 (23 ± 2) h	6.3 ± 1.9	8.7 ± 2.3	8.2 ± 2.4	11.7 ± 2.0
TP3 (47 ± 2) h	TP3 (47 ± 2) h	6.4 ± 2.0	8.6 ± 2.4	7.9 ± 2.6	10.9 ± 3.3
TP3 (47 ± 2) h	TP4 (71 ± 2) h	6.5 ± 2.0	8.5 ± 2.3	6.3 ± 3.4	7.3 ± 7.0
TP3 (47 ± 2) h	TP5 (167 ± 2) h	6.6 ± 1.9	8.6 ± 2.3	13.6 ± 4.4	18.0 ± 3.9
TP4 (71 ± 2) h	TP1 (2.5 ± 0.9) h	9.8 ± 2.5	13.7 ± 3.4	14.9 ± 5.1	19.3 ± 5.4
TP4 (71 ± 2) h	TP2 (23 ± 2) h	9.8 ± 2.5	13.6 ± 3.4	7.8 ± 2.4	10.8 ± 2.1
TP4 (71 ± 2) h	TP3 (47 ± 2) h	9.7 ± 2.5	13.4 ± 3.5	7.4 ± 2.6	10.2 ± 3.4
TP4 (71 ± 2) h	TP4 (71 ± 2) h	10.0 ± 2.5	13.6 ± 3.4	6.2 ± 3.4	7.1 ± 7.0
TP4 (71 ± 2) h	TP5 (167 ± 2) h	9.8 ± 2.5	13.5 ± 3.4	13.3 ± 4.4	18.0 ± 3.9
TP5 (167 ± 2) h	TP1 (2.5 ± 0.9) h	12.5 ± 3.1	16.6 ± 4.6	14.9 ± 5.1	19.3 ± 5.3
TP5 (167 ± 2) h	TP2 (23 ± 2) h	12.5 ± 3.1	16.5 ± 4.6	7.9 ± 2.4	11.0 ± 2.0
TP5 (167 ± 2) h	TP3 (47 ± 2) h	12.3 ± 3.1	16.1 ± 4.8	8.0 ± 2.6	10.7 ± 3.3
TP5 (167 ± 2) h	TP4 (71 ± 2) h	12.5 ± 3.1	16.2 ± 4.7	6.2 ± 3.4	7.2 ± 7.0
TP5 (167 ± 2) h	TP5 (167 ± 2) h	12.6 ± 3.1	16.4 ± 4.6	13.6 ± 4.4	18.0 ± 3.9

Table S4. Performance metrics of Scenario 4. Mean absolute percentage error (MAPE) and root-mean-square error (RMSE) of single-time-point (STP) dosimetry in cycle 2 (C2) with all-time-point (ATP) fitting in cycle 1 (i.e., five time points), thereby taking into account the inter-cycle variability as defined in Eq. (2).

Time Point	MAPE (%)	RMSE (%)
TP1 (2.5 ± 0.9) h	13.9 ± 5.0	17.2 ± 6.0
TP2 (23 ± 2) h	7.8 ± 2.4	11.1 ± 2.0
TP3 (47 ± 2) h	7.8 ± 2.6	10.7 ± 3.4
TP4 (71 ± 2) h	6.7 ± 3.4	7.5 ± 7.0
TP5 (167 ± 2) h	13.3 ± 4.6	17.7 ± 4.2

Table S5. Performance metrics for the Hänscheid method.[3] The values represent the mean absolute percentage error (MAPE) and root-mean-square error (RMSE) for single-time-point (STP) dosimetry evaluated at each time point within the single-cycle dataset. The earliest time point, TP1 (2.5 ± 0.9) h, and the latest time point, TP5 (167 ± 2) h after injection, were excluded from this evaluation because they fall outside the effective interval for this specific method.[3] Uncertainties were not reported because this method did not include uncertainty estimates.

Time Point	Cycle 1		Cycle 2	
	MAPE (%)	RMSE (%)	MAPE (%)	RMSE (%)
TP2 (23 ± 2) h	22.0	22.4	20.5	22.1
TP3 (47 ± 2) h	13.0	14.6	16.0	18.0
TP4 (71 ± 2) h	19.2	22.0	19.4	21.4

Table S6. Performance metrics for the Madsen method.[4] The values represent the mean absolute percentage error (MAPE) and root-mean-square error (RMSE) for single-time-point (STP) dosimetry evaluated at each time point within the single-cycle dataset. Uncertainties were not reported because this method did not include uncertainty estimates.

**Time Point**	**Cycle 1**		**Cycle 2**	
	**MAPE (%)**	**RMSE (%)**	**MAPE (%)**	**RMSE (%)**
TP1 (2.5 ± 0.9) h	32.5	43.2	22.3	25.2
TP2 (23 ± 2) h	5.3	6.2	10.7	14.5
TP3 (47 ± 2) h	17.0	18.2	20.0	21.5
TP4 (71 ± 2) h	21.0	23.3	21.2	23.0
TP5 (167 ± 2) h	19.6	22.1	30.7	41.2

Figures

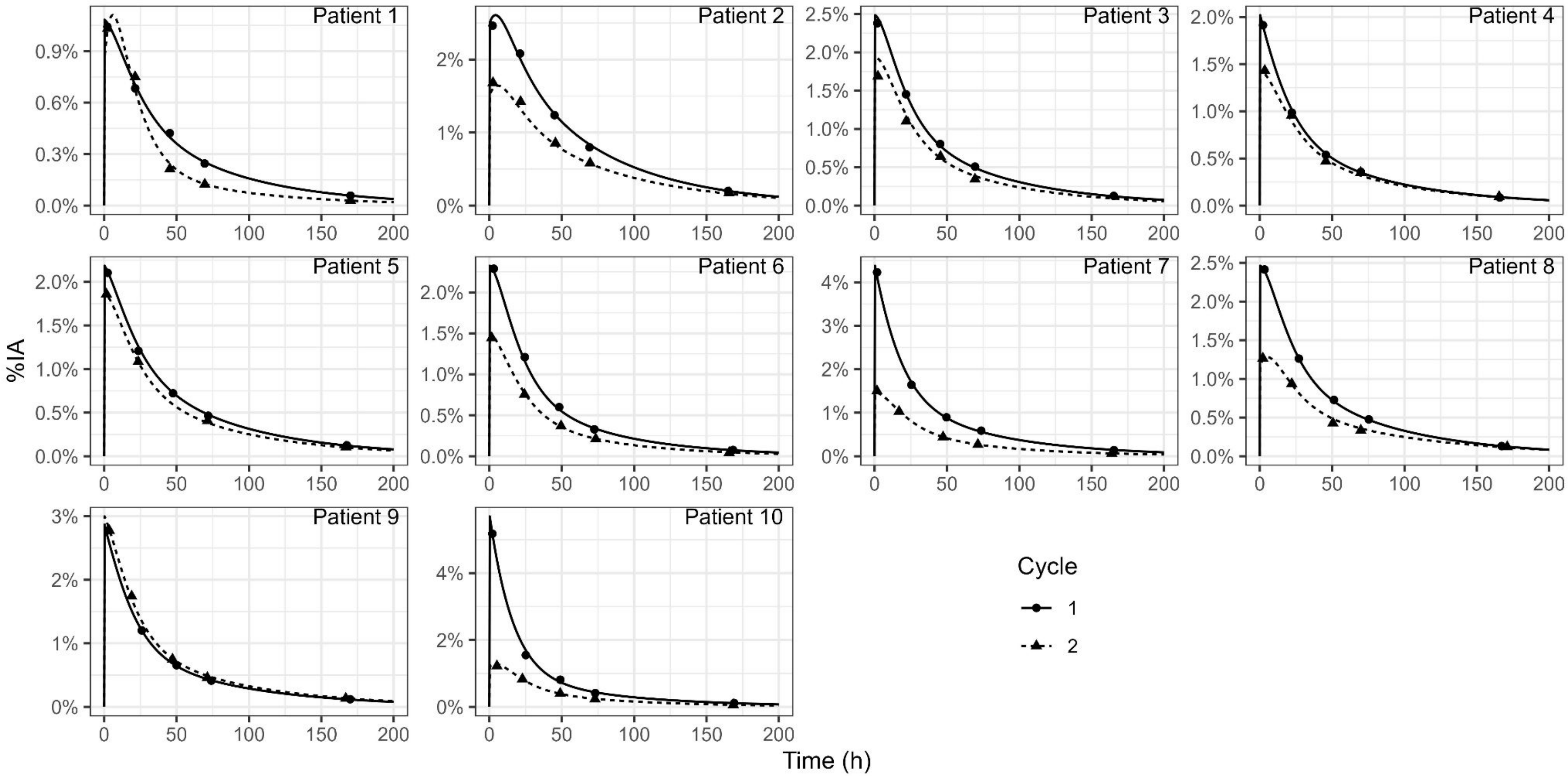

Figure S1. Reference biokinetic curves for the right kidneys with all-time-point (ATP) fitting, considering the variability defined in Eq. (2).

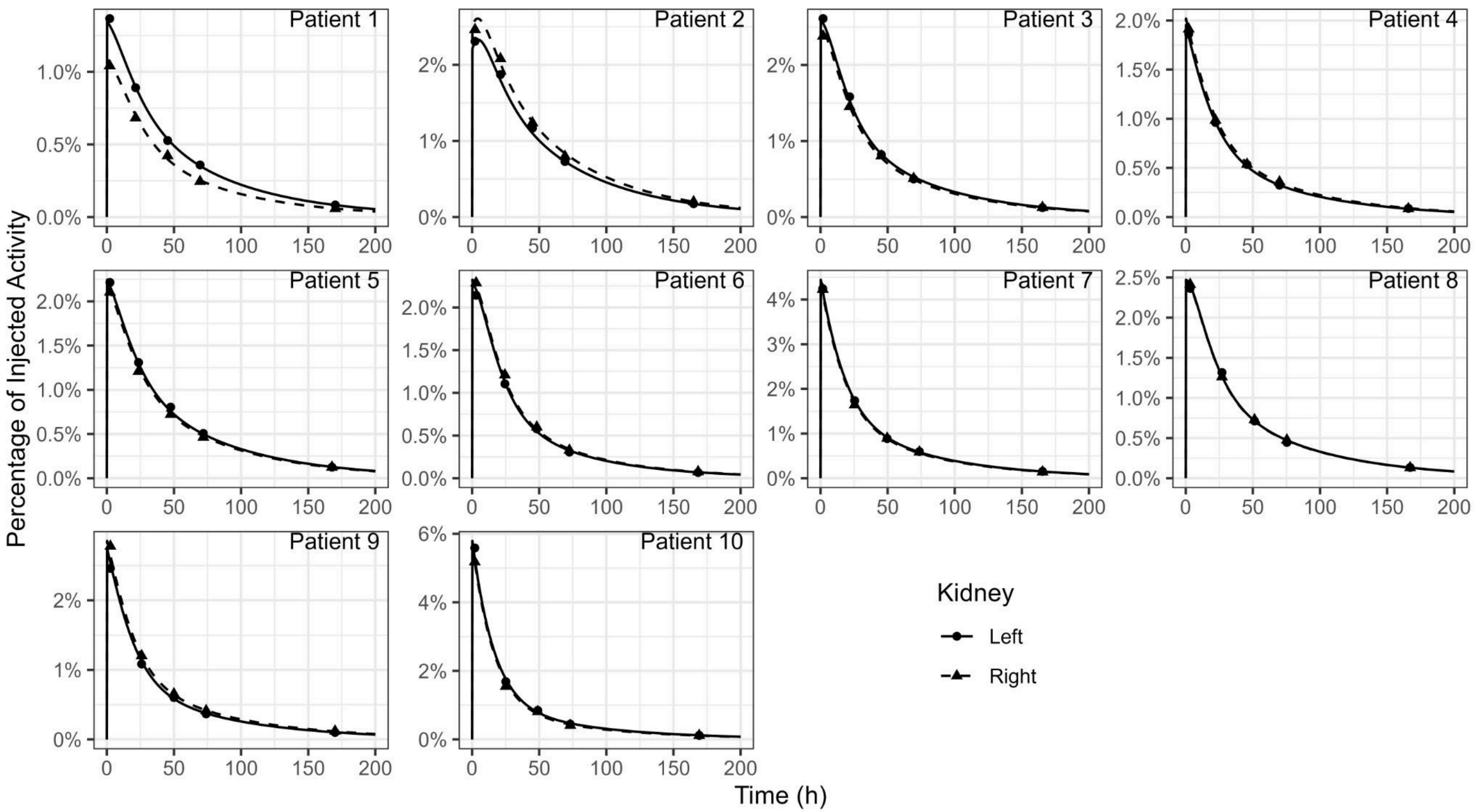

Figure S2. Biokinetic curves showing the percentage of injected activity over time for the left and right kidneys during the first cycle of [^{177}Lu]Lu-PSMA-617 therapy.

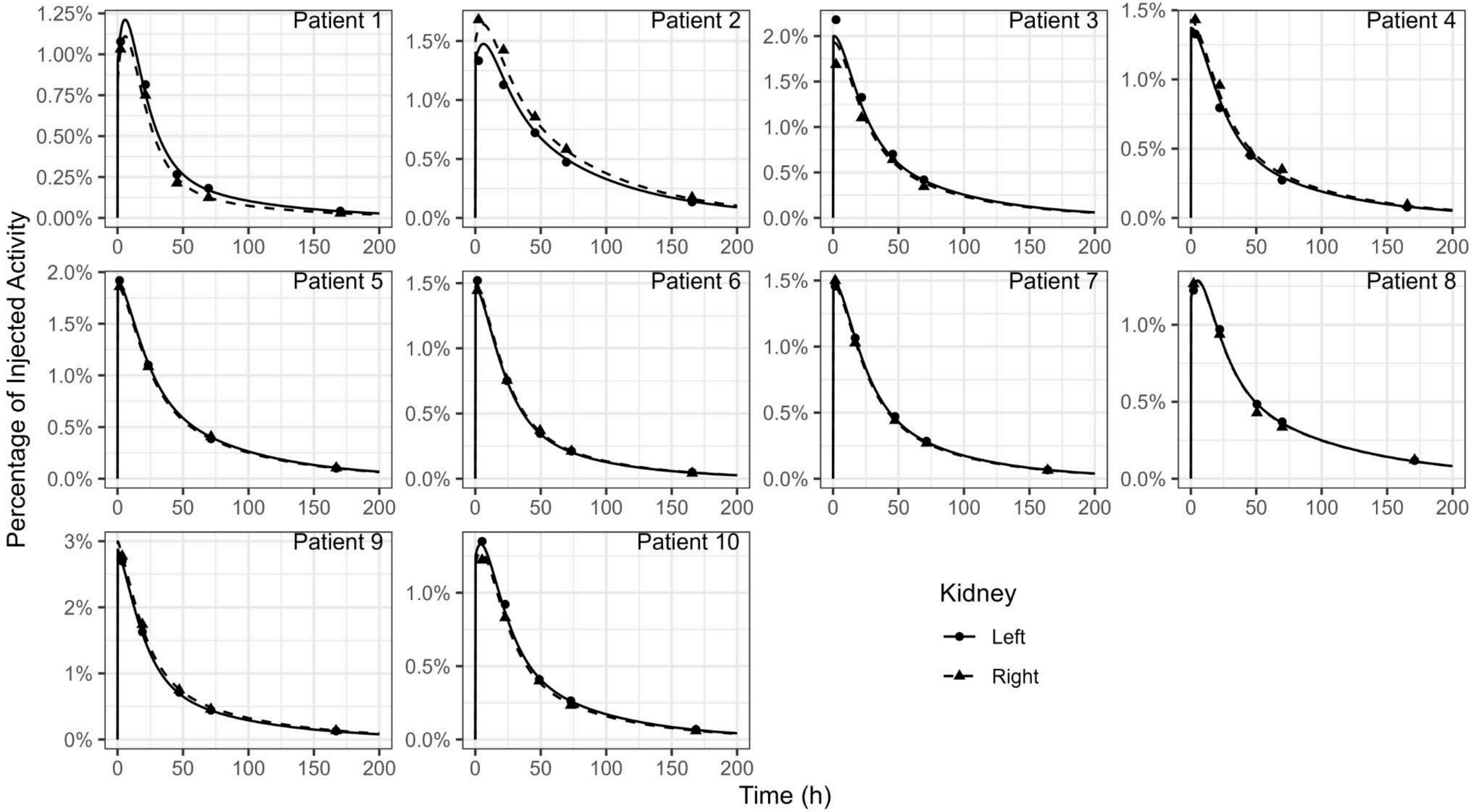

Figure S3. Biokinetic curves showing the percentage of injected activity over time for the left and right kidneys during the second cycle of [^{177}Lu]Lu-PSMA-617 therapy.

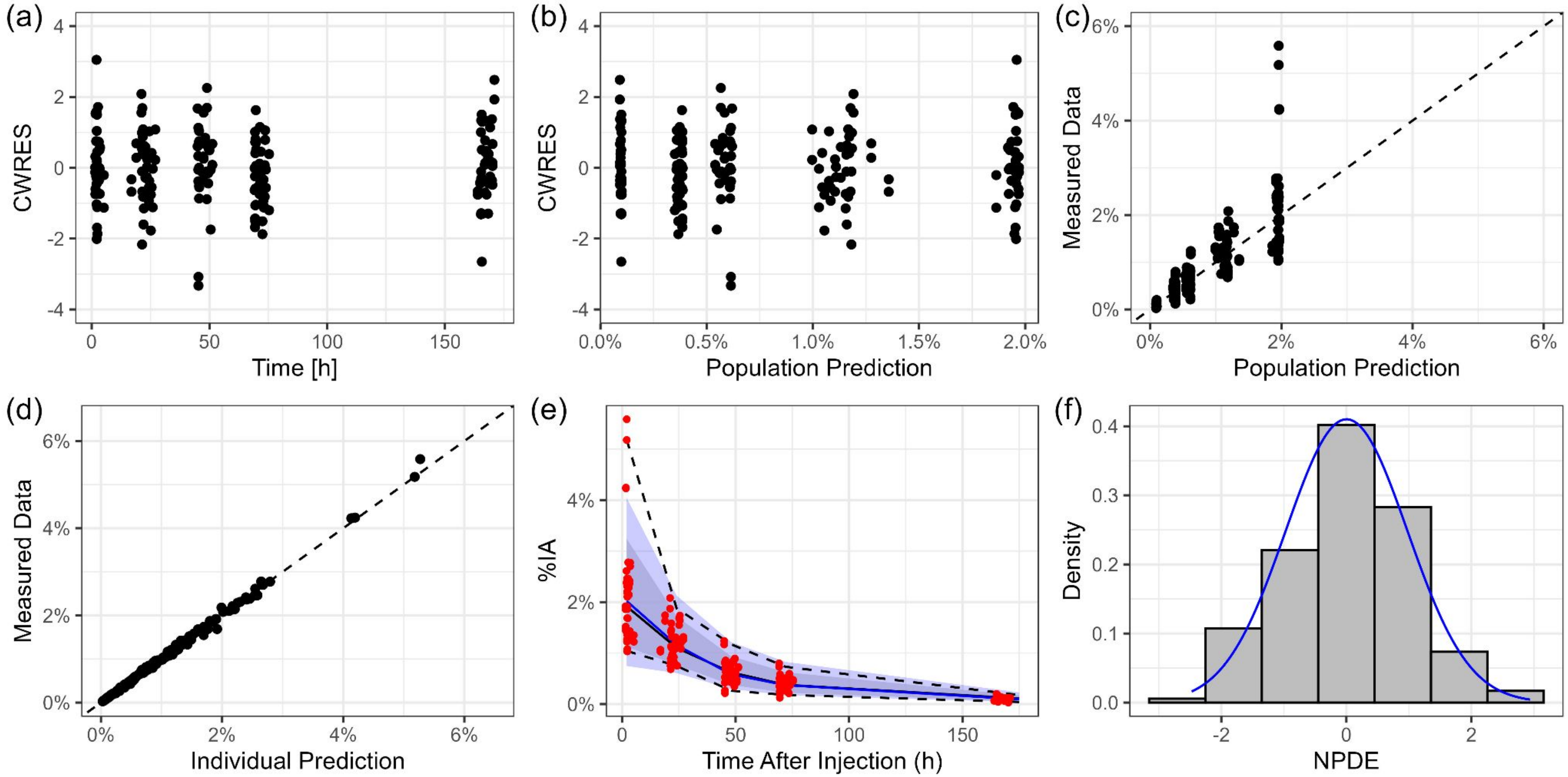

Figure S4. Nonlinear mixed-effects (NLME) diagnostic plots for evaluating model adequacy. (a) Conditional weighted residuals (CWRES)-versus-time plots show standardized residuals over time. The scatter is approximately normally distributed around zero with no systematic time-dependent bias, indicating adequate description of the all-time-point (ATP) dataset. (b) CWRES versus population prediction evaluates model performance across the whole prediction range. The CWRES are symmetrically distributed around zero without systematic bias, suggesting the model is adequate across the entire range of data values. (c) Observed data versus population prediction, i.e., prediction using only fixed effects, ideally shows normally distributed scatter around the line $y = x$ (identity line). The scatter aligns reasonably well with the line of identity, with no major systematic deviation, suggesting that the residual-error model is appropriate. (d) Observed data versus individual predictions (incorporating individual random effects into the model). The scatter was close to the identity line, suggesting that the random effects improve the model fit to the data. (e) Visual predictive check (VPC) based on 1,000 simulations. Red dots represent observed data, and shaded regions depict the 2.5th and 97.5th percentile. Median lines (observed data: black; simulated data: blue) overlap well, and most observed data fall within the prediction intervals, demonstrating good predictive performance. (f) Normalized prediction distribution errors (NPDE). The histogram of NPDE approximates the expected theoretical distribution (blue line), indicating that predictive discrepancies are within acceptable limits.

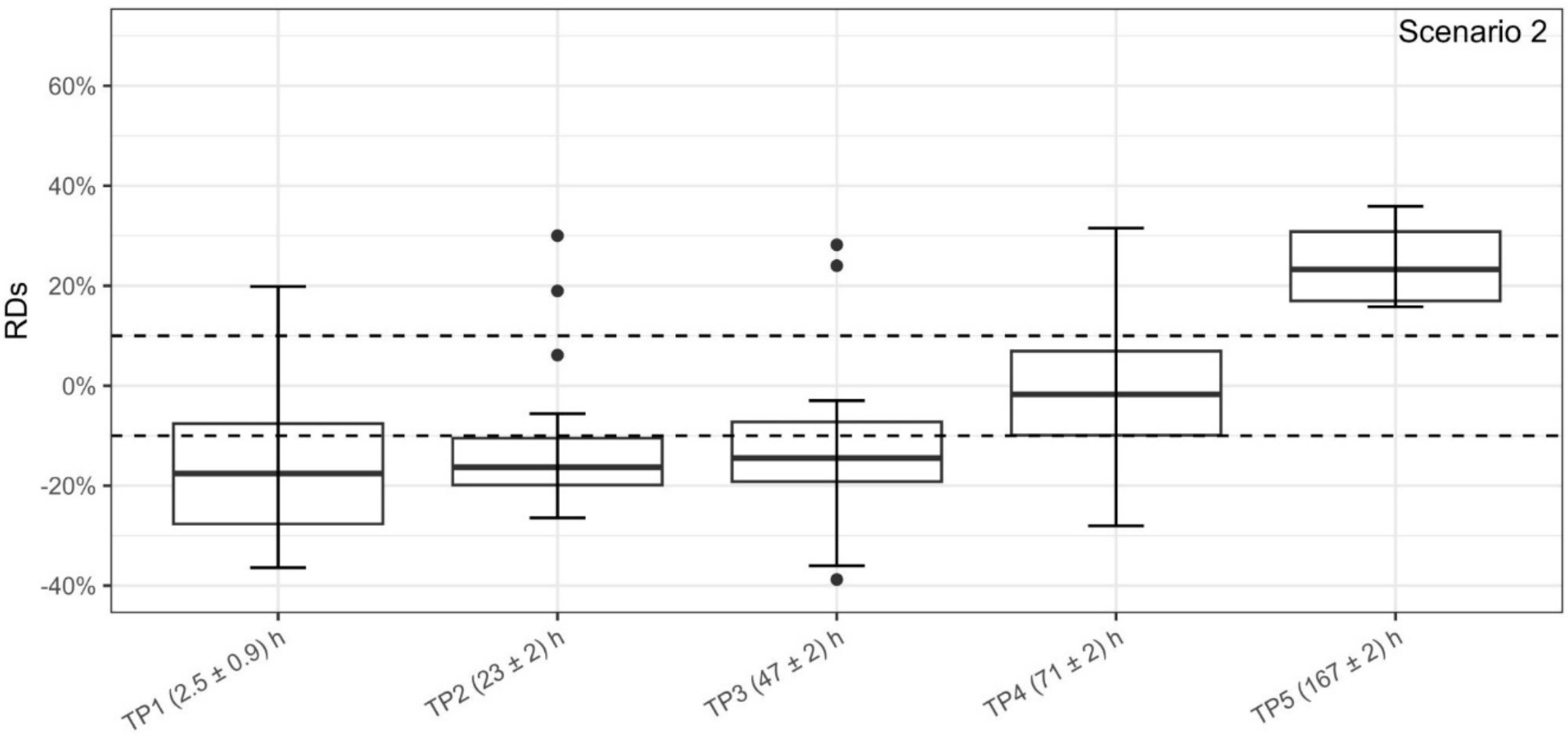

Figure S5. Relative deviations (RDs) of single-time-point (STP) dosimetry in the second cycle (C2) using Scenario 2, where the time-integrated activity coefficients (TIACs) in the second cycle were estimated using a monoexponential fit function with the effective half-life derived from all-time-point (ATP) fitting in the first cycle (C1) dataset, as reported in the literature.[2] TP4 (71 ± 2) h and TP5 (167 ± 2) h after injection yielded 23 extreme RD values up to ~5000% (not shown). Horizontal dashed lines indicate an absolute RD of 10%.

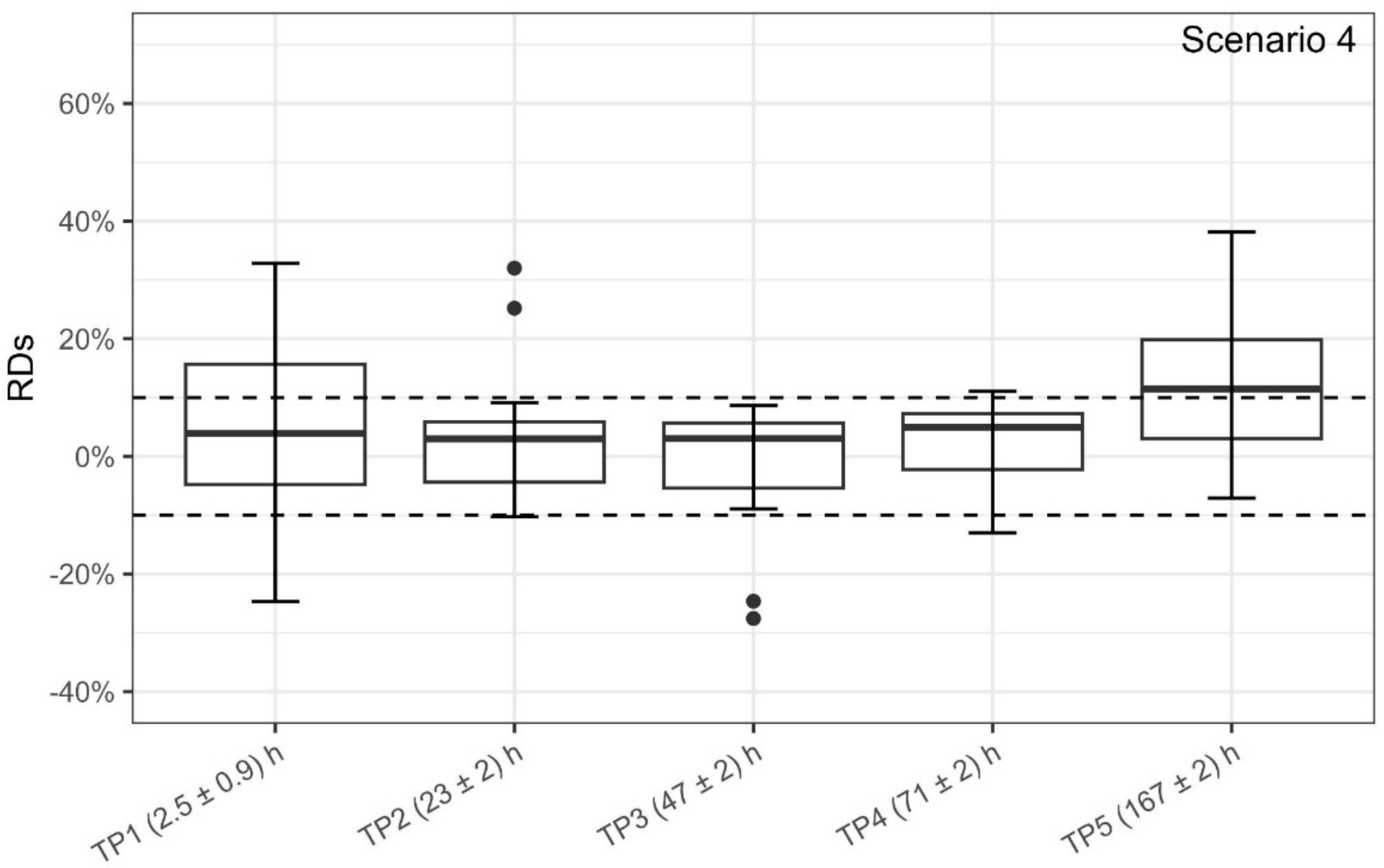

Figure S6. Relative deviations (RDs) of single-time-point (STP) dosimetry in the second cycle (C2) across time points, derived from Scenario 4: all-time-point (ATP) dosimetry in the first cycle (C1) and STP dosimetry in C2. Horizontal dashed lines indicate an absolute RD of 10%.

References

1. Budiansah I, Hardiansyah D, Riana A, Pawiro SA, Beer AJ, Glatting G. Accuracy and precision analyses of single-time-point dosimetry utilising physiologically-based pharmacokinetic modelling and non-linear mixed-effects modelling. *EJNMMI Phys*. 2025;12(1):26. doi:10.1186/s40658-025-00726-7

2. Willowson KP, Eslick E, Ryu H, Poon A, Bernard EJ, Bailey DL. Feasibility and accuracy of single time point imaging for renal dosimetry following ^{177}Lu-DOTATATE ('Lutate') therapy. *EJNMMI Phys*. 2018;5(1):33. doi:10.1186/s40658-018-0232-9

3. Hänscheid H, Lapa C, Buck AK, Lassmann M, Werner RA. Dose Mapping After Endoradiotherapy with ^{177}Lu-DOTATATE/DOTATOC by a Single Measurement After 4 Days. *JNM*. 2018;59(1):75-81. doi:10.2967/jnumed.117.193706

4. Madsen MT, Menda Y, O'Dorisio TM, O'Dorisio MS. Technical Note: Single time point dose estimate for exponential clearance. *Med Phys*. 2018;45(5):2318-2324. doi:10.1002/mp.12886
